# Rrp17p Is a Eukaryotic Exonuclease Required for 5′ End Processing of Pre-60S Ribosomal RNA

**DOI:** 10.1016/j.molcel.2009.11.011

**Published:** 2009-12-11

**Authors:** Marlene Oeffinger, Daniel Zenklusen, Angelica Ferguson, Karen E. Wei, Aziz El Hage, David Tollervey, Brian T. Chait, Robert H. Singer, Michael P. Rout

**Affiliations:** 1 Rockefeller University, New York, NY 10065, USA; 2 Department of Anatomy and Structural Biology, Albert Einstein College of Medicine, Bronx, NY 10461, USA; 3 Wellcome Trust Centre for Cell Biology, University of Edinburgh, Edinburgh EH9 3JR, UK

**Keywords:** RNA, PROTEINS

## Abstract

Ribosomal processing requires a series of endo- and exonucleolytic steps for the production of mature ribosomes, of which most have been described. To ensure ribosome synthesis, 3′ end formation of rRNA uses multiple nucleases acting in parallel; however, a similar parallel mechanism had not been described for 5′ end maturation. Here, we identify Rrp17p as a previously unidentified 5′–3′ exonuclease essential for ribosome biogenesis, functioning with Rat1p in a parallel processing pathway analogous to that of 3′ end formation. Rrp17p is required for efficient exonuclease digestion of the mature 5′ ends of 5.8S_S_ and 25S rRNAs, contains a catalytic domain close to its N terminus, and is highly conserved among higher eukaryotes, being a member of a family of exonucleases. We show that Rrp17p binds late pre-60S ribosomes, accompanying them from the nucleolus to the nuclear periphery, and provide evidence for physical and functional links between late 60S subunit processing and export.

## Introduction

Biogenesis of eukaryotic ribosomes is a highly coordinated process that largely takes place in the nucleolus. Work by many groups has defined the processing pathway for ribosomal RNA (rRNA) and identified ∼170 proteins involved in ribosome biogenesis, while proteomic analyses have provided a roughly outlined pathway of preribosome assembly in *Saccharomyces cerevisiae* (Fatica and Tollervey, 2002; Venema and Tollervey, 1999). Three of the four rRNAs (18S, 5.8S, and 25/28S rRNA) are derived from the 35S rRNA precursor transcribed by RNA polymerase I, while the fourth rRNA (5S rRNA) is transcribed separately by RNA polymerase III. The 35S pre-rRNA is packaged into a 90S ribonucleoprotein particle (RNP) together with a subset of assembly factors and ribosomal proteins (r-proteins). After undergoing extensive site-specific modifications, internal and external spacer regions (ITS and ETS) are removed (Figure S1) by an ordered series of endonucleolytic cleavages and exonucleolytic digestion steps to form functional 40S and 60S ribosomal subunits (Venema and Tollervey, 1999).

A large number of studies have made it clear that, apart from the r-proteins, a multitude of *trans*-acting, nonribosomal factors play a crucial role in the assembly of functional eukaryotic ribosomes (Fatica and Tollervey, 2002; Fromont-Racine et al., 2003). Although the precise function of many of these factors is still unknown, recent data have shown that some of them determine separate but parallel-acting assembly and processing routes that ensure maturation of preribosomes through a network of redundant parallel pathways (Pérez-Fernández et al., 2007). One such example of a parallel pathway is the maturation of 5.8S rRNA. Roughly 80% of 27SA_2_ pre-rRNA is cleaved at site A_3_ by RNase MRP, and the resulting 27SA_3_ precursor is processed exonucleolytically into 27SB_S_ pre-rRNA; however, the remaining 20% are converted into the 27SB_L_ pre-rRNA by an endonucleolytic cleavage (Figure S1) (Venema and Tollervey, 1999). As a result, two mature 5.8S rRNA species (5.8S_L_ [long] and 5.8S_S_ [short]) are produced, in a ratio of about 1:5.

The 5′ end maturation of 5.8S_S_ (and 25S rRNA) in yeast has been attributed to the nuclear 5′–3′ exonuclease Rat1p and its cofactor Rai1p (El Hage et al., 2008; Henry et al., 1994). Its cytoplasmic homolog Xrn1p is involved in the degradation of aberrant rRNA precursors, and its absence does not affect the ratio of 5.8S L:S rRNAs (El Hage et al., 2008; Johnson, 1997). However, defects in both 5.8S_S_ and 25S rRNA maturation were exacerbated by deletion of *XRN1* in cells depleted for Rat1p, indicating that Xrn1p could take over rRNA maturation steps in its absence (El Hage et al., 2008; Geerlings et al., 2000; Henry et al., 1994). While Rat1p seems to be the only 5′–3′ exonuclease implicated in the maturation of 5′ ends, 3′ end formation of 5.8S rRNA involves multiple nucleases that act sequentially to ensure correct and precise trimming of the pre-rRNA (Briggs et al., 1998; de la Cruz et al., 1998; Faber et al., 2002; Mitchell et al., 1996). In the absence of any one of these nucleases, 3′ end maturation still occurs, albeit not as efficiently (Allmang et al., 2000; de la Cruz et al., 1998). This ensures that even in the absence of one of these nucleases, production of mature ribosomal subunits still continues. However, a similarly redundant mechanism involving multiple nucleases had not been uncovered for 5′ end maturation of 5.8S and 25S rRNA. Here, we describe the characterization of the yeast protein Ydr412p as a highly conserved exonuclease that is required for the 5′ end maturation of 5.8S and 25S rRNAs, demonstrating that 5′ end processing also has a redundant pathway.

## Results

### Rrp17p Is Part of NPC-Associated Pre-60S Ribosomal Subunits

We have previously described a method for purifying yeast nuclear pore complexes (NPCs). This NPC fraction was highly enriched in proteins known to be associated with late processing and export of mRNA and ribosomal subunits, likely caught during the process of chaperoning these cargoes to and through the NPC (Rout et al., 2000). One of these NPC-associated proteins caught our attention; this protein, Ydr412p, had no previously assigned function, yet localized to the nucleolus, as does its human homolog, Nol12/Nop25 (Huh et al., 2003; Suzuki et al., 2006). Its association with the NPC and with the nucleolus suggested that it might be a late-acting ribosome synthesis factor. As discussed below, this hypothesis proved correct, and we have thus termed it a member of the *r*ibosomal *R*NA *p*rocessing family, Rrp17p.

**Figure 1:**
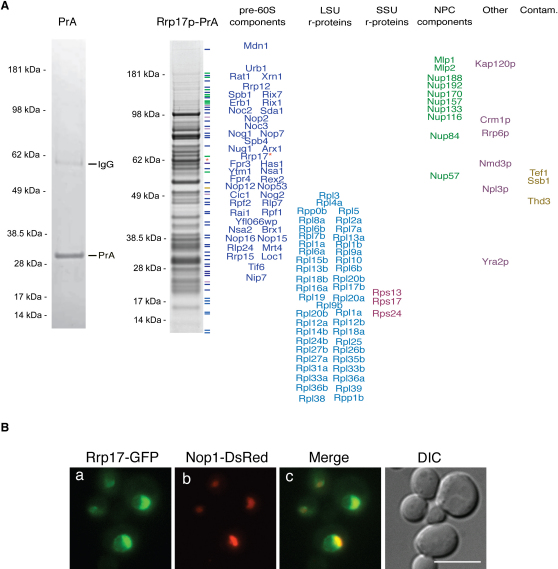
Isolated Rrp17p-Associated Complexes Contain Pre-60S Ribosomal and Nuclear Peripheral Components (A) Left: Protein A (PrA) was expressed under the control of the endogenous *ZPR1* promoter and affinity purified using IgG-conjugated magnetic beads (Oeffinger et al., 2007). Right: Rrp17p-associated complexes were affinity purified via the PrA tag. Proteins associated with the isolated tagged complexes were resolved by SDS-PAGE and visualized by staining with Coomassie blue. Proteins identified by mass spectrometry are listed on the right. (B) Rrp17p is a nucleolar protein. A Rrp17-GFP strain, coexpressing the nucleolar marker DsRedNop1p, was examined for localization of Rrp17p in live cells. Bar represents 10 μm.

To better understand the function of Rrp17p, we isolated a genomically tagged version of the protein ex vivo to determine the proteins with which it interacts, using our fast single-step affinity purification procedure (Oeffinger et al., 2007). We identified 109 proteins in the isolated Rrp17p complex. Ten of these were NPC components, consistent with the association of Rrp17p with NPCs (Rout et al., 2000). We also found 48 ribosomal proteins, 45 of which were components of the large subunit, and 43 known preribosomal processing factors involved in late steps in 60S subunit maturation (Figure 1A, right) (Bassler et al., 2001; Fatica et al., 2002; Nissan et al., 2002). Moreover, we were able to isolate several transient components, such as nucleoporins, the pre-60S subunit export factor Crm1p, and the exonucleases Rat1p, Xrn1p, and Rrp6p (Henry et al., 1994; Johnson et al., 2002). This is consistent with a role for Rrp17p in ribosomal maturation and/or export. In addition, we also dentified Npl3p and Yra2p. Depletion of Npl3p (Nop3p) affects ribosome synthesis (Russell and Tollervey, 1992), but no role for Yra2p in ribosome synthesis has so far been reported.

To confirm the association of Rrp17p with preribosomes, we performed sucrose gradient centrifugation on whole-cell lysates from cells expressing Rrp17-Protein A (PrA) and compared the sedimentation of Rrp17-PrA to that of the pre-rRNAs of both the 60S and 40S ribosomal subunits (Figure S2A). Rrp17-PrA cosediments with 27SB pre-RNA, a component of pre-60S subunits, but not with 20S pre-rRNA, the precursor to mature 18S rRNA and component of 40S preribosomes. We also determined which rRNA precursors were associated with the isolated Rrp17-PrA containing complexes. Coisolation was seen for late pre-60S RNA components with Rrp17-PrA complexes compared to mock purifications (Figures S2B and S2Cb-S2Ce), while no 90S (35S pre-rRNA) (Figure S2Ca) or 40S components (18S rRNA) (Figure S2D) were detected. Taken together, these data demonstrate that Rrp17p associates only with pre-60S ribosomal subunits.

Using a functional GFP-tagged allele, we assessed whether the in vivo cellular localization of Rrp17p is consistent with an association with preribosomes. Rrp17p is concentrated mainly in the nucleolus, with some residual nuclear staining (Figure 1B). This same pattern is found for numerous proteins known to associate with late preribosomes (Huh et al., 2003). We also noted a faint punctate signal at the nuclear periphery, which is consistent with the association of Rrp17-GFP with the NPC. Taken together, these data indicate that Rrp17p is a bona fide component of pre-60S ribosomal subunit that associates with the complexes in the nucleolus and accompanies them to the NPC prior to their export.

### Rrp17p Is Involved in 5′ End Maturation of 60S Subunit rRNAs

**Figure 2:**
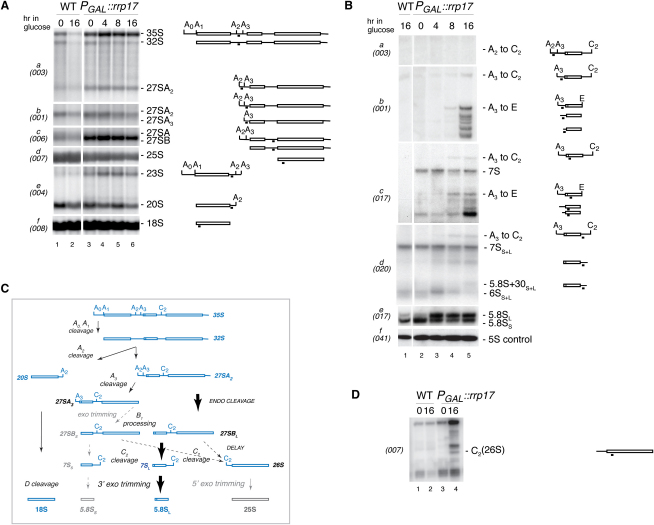
Rrp17p Depletion Affects Maturation of 60S RNA Components (A) Northern analysis of high-molecular-weight RNA. RNA was extracted from wild-type and *P_GAL_::rrp17* strains during growth on permissive raffinose/galactose/sucrose-containing medium and after transfer to glucose medium. Pre-rRNAs are indicated schematically on the right. Rectangles represent the mature rRNA and thin lines the transcribed spacers, with the position of the probe used underlined. Probe names are indicated in parentheses on the left. (B) Northern analysis of low-molecular-weight RNA extracted and represented as indicated for (A). (C) rRNA processing pathway depicting the affects of Rrp17p depletion on synthesis of 5.8S and 25S rRNAs (bold arrows, favored pathways; dashed arrows, disrupted pathways). 5.8S rRNA synthesis has shifted from the major short to the minor long form; 26S to 25S rRNA conversion is carried out inefficiently. (D) Primer extension analysis using a primer complementary to the 5′ region of 25S rRNA. The pre-rRNA corresponding to the identified stops is indicated in parentheses on the right.

**Figure 3:**
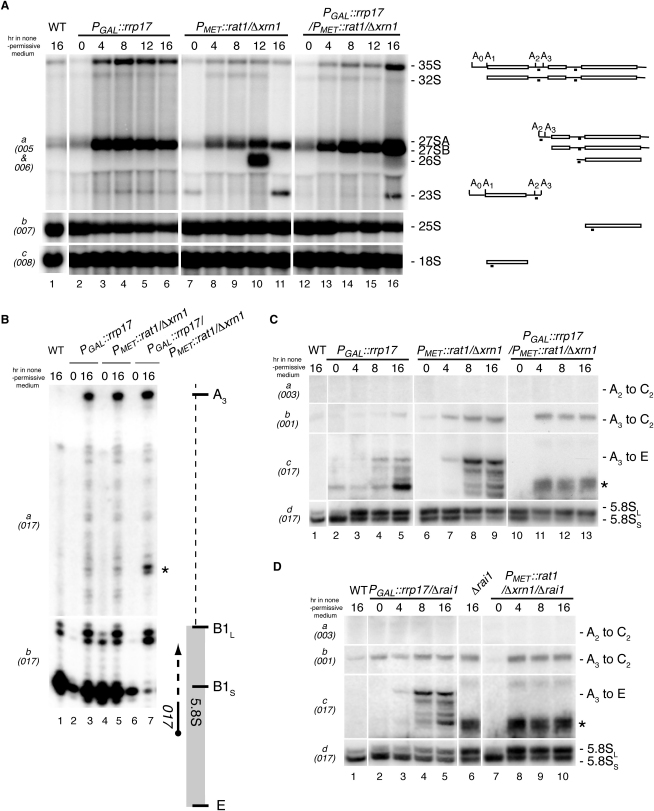
Rrp17p Is Needed for Efficient 5′ End Formation of 5.8S_S_ and 25S rRNAs (A) Northern analysis of high-molecular-weight RNA. RNA was extracted from wild-type, *P_GAL_::rrp17*, *P_MET_::rat1/xrn1Δ*, and *P_GAL_::rrp17/P_MET_::rat1/xrn1Δ* strains during growth on permissive synthetic dropout medium and after transfer to glucose containing medium ±5 mM methionine for the times indicated. (B) Primer extension using a primer complementary to the 5′ region of 5.8S rRNA. Pre-rRNAs are indicated schematically on the right. (C) Northern analysis of low-molecular-weight RNA extracted as described for (A). (D) Northern analysis of low-molecular-weight RNA. RNA was extracted from wild-type, *P_GAL_::rrp17/rai1Δ*, *rai1Δ*, and *P_MET_::rat1/xrn1Δ/rai1Δ* strains during growth on permissive synthetic dropout medium and after transfer to glucose synthetic dropout medium ±5 mM methionine for the times indicated. ^∗^ denotes putative endonucleolytic cleavage product.

Since Rrp17p was associated with late 60S preribosomes, we examined its possible function during 60S maturation. Deletion of *RRP17* was shown to be lethal (Giaever et al., 2002). Hence, we studied the effect of depletion of Rrp17p in a strain expressing an HA-Rrp17p fusion construct under the control of the repressible *GAL1* promoter at the endogenous *RRP17* locus. Growth of isogenic wild-type and *P_GAL_::rrp17* strains was identical in galactose-containing medium. However, growth of the *P_GAL_::rrp17* strain declined progressively following transfer to glucose medium (Figures S3A and S3B). Northern hybridization analysis showed mild accumulation of 35S and 23S pre-rRNAs (Figure 2Ae), the latter being a product of premature cleavage of 32S pre-rRNA at site A_3_. The 27SA_3_ pre-rRNA also accumulated in Rrp17p-depleted cells (Figure 3B, lanes 1-3), as well as 27SB pre-rRNA, while mature 25S rRNA was reduced ∼3-fold after 16 hr in glucose, compared to wild-type and 18S rRNA levels (Figures 2Ac, 2Ad, and S4A). In addition, primer extension analysis showed a moderate increase of 26S pre-rRNA, the 5′-extended precursor of 25S rRNA and product of C_2_ cleavage (Figure 2D). In wild-type cells, 26S pre-rRNA is processed very rapidly by the 5′–3′ exonucleases Rat1p and Xrn1p and is not usually detected (Geerlings et al., 2000). However, accumulation of 27SB and 26S precursors suggested a delay in both cleavage at site C_2_ and subsequent processing of 26S (Figure 2C). No alterations were seen in the levels of 20S pre-rRNA and mature 18S rRNA (Figures 2Ae and 2Af).

Analysis of the 5.8S rRNA synthesis pathway following transfer to glucose showed no accumulation of 7S pre-rRNA in the absence of Rrp17p, but a minor accumulation of 3′-extended intermediates indicated that 3′ end processing of 5.8S rRNA was less efficient (Figure 2Bd). Moreover, 5′-extended forms of 7S pre-rRNA (A_3_ to C_2_; A_3_ to E) were detected (Figures 2Bb–2Bd), indicating that processing in ITS2 occurred prior to that in ITS1, a phenotype that has previously been observed when processing in ITS1 was slowed (Eppens et al., 2002; Faber et al., 2004; Venema and Tollervey, 1999). In addition, the Rrp17p-depleted strain showed a clear alteration in the alternative processing pathways that generate the mature 5.8S_L_ and 5.8S_S_ rRNAs (Figure 2Be, lanes 3–5), in which more 5.8S_L_ was found than in wild-type cells (Figure 2Be, lane 1). Accumulated aberrant 5′-extended forms of 5.8S rRNA were also observed, indicating inefficient exonucleolytic maturation of 5.8S_S_ rRNA and thus a delay in ITS1 processing (Figures 2Bb and 2Bc). Synthesis of 5S rRNA was not affected (Figure 2Bf).

A switch from 5.8S_S_ to 5.8S_L_ rRNA was previously observed only in mutants that impair either cleavage at A_3_ (Rrp5p or RNase MRP) or subsequent 5′ exonuclease processing to site B1_S_, the 5′ end of mature 5.8S_S_ rRNA (Rat1p and Xrn1p) (El Hage et al., 2008; Eppens et al., 1999; Faber et al., 2006; Henry et al., 1994). Taken together, these data suggest that Rrp17p is required for efficient exonuclease digestion to the mature 5′ ends of 5.8S_S_ and 25S RNAs (Figure S1). The delay in early processing at the sites A_0_, A_1_, and A_2_ is probably secondary, as similar effects have previously been described for several other factors, though their basis is not fully understood (Venema and Tollervey, 1999).

### Rrp17p Is Involved in 5.8S_S_ Maturation Parallel to Processing by Rat1p

The formation of the 5′ end of both 5.8S_S_ and 25S rRNAs has previously been attributed to the 5′–3′ exonucleases Rat1p and Xrn1p (Henry et al., 1994). However, deletion of the nonessential Xrn1p alone did not lead to detectable 5′ end processing defects, while depletion of the essential nuclear Rat1p exhibited significant 5.8S and 25S maturation defects, which were exacerbated by deletion of *XRN1* in this background, indicating that Xrn1p function in 5′ end processing is seen only in the absence of Rat1p (El Hage et al., 2008; Geerlings et al., 2000; Henry et al., 1994). Hence, we focused on potential links between the functions of Rrp17p and Rat1p during 5.8S rRNA maturation in an *xrn1Δ* background.

We constructed a *P_GAL_::rrp17/P_MET_::rat1/xrn1Δ* strain and compared synthesis of 25S and 5.8S rRNA to *P_GAL_::rrp17* and *P_MET_::rat1/xrn1Δ* strains. Analysis of the triple mutant under restrictive conditions revealed a strong accumulation of both 27SA, specifically 27SA_3_ (Figure 3Ba), and 27SB pre-rRNAs and a decrease in mature 25S rRNA (Figure 3A, right a and b), whereas maturation of 18S rRNA was not affected (Figure 3A, right c). This suggests that processing in both ITS1 and ITS2 was delayed. In comparison, less accumulation of 27S precursors was observed in the *P_MET_::rat1/xrn1Δ* mutant under restrictive conditions, but instead, a substantial increase of 26S was observed (Figure 3A, middle a) (Geerlings et al., 2000); after 16 hr, no more 26S was detected, while levels of mature 25S rRNA remained normal. This points toward the presence of another exonuclease, taking over in the absence of both Rat1p and Xrn1p to ensure production of mature 25S rRNA. Given the phenotype of the *P_GAL_::rrp17/P_MET_::rat1/xrn1Δ* strain, it is conceivable that Rrp17p may be this exonuclease. However, a potential fourth nuclease does not appear to be involved, as 25S rRNA levels reduce over time under restrictive conditions in the *P_GAL_::rrp17/P_MET_::rat1/xrn1Δ* strain (Figure 3A, right b).

As previously shown, 5′-extended forms of 5.8S rRNA accumulated in *P_MET_::rat1/xrn1Δ* cells when Rat1p was depleted (Figures 3B, lane 5, and 3C, lanes 7–9) (El Hage et al., 2008); similar 5′-extended forms were also observed in the *P_GAL_::rrp17* strain (Figures 3B, lane 3, and 3C, lanes 3–5), but not in the wild-type (Figures 3B lane 1, and 3C, lane 1), in restrictive medium. In contrast, the 5′ end-extended forms were not present in *P_GAL_::rrp17/P_MET_::rat1/xrn1Δ* cells when both proteins were depleted. Instead, a drastic drop in mature 5.8S_S_ rRNA and a switch to the 5.8S_L_ rRNA was observed, together with the appearance of a single 5′-extended RNA (Figures 3B, lane 7, and 3C, lanes 11–13). This particular 5′-extended species is believed to be generated by an endonucleolytic cleavage in ITS1 at the base of the stem that lies 5′ to site B1_L_ (El Hage et al., 2008). In all three strains, precursors were not extended to site A_2_, indicating that cleavage at A_3_ had occurred (Figure 3Ca). The appearance of an A_3_–C_2_ fragment, however, indicates that processing in ITS2 occurs prior to ITS1, and 3′ end processing of 5.8S rRNA was also less efficient in the *P_GAL_::rrp17/P_MET_::rat1/xrn1Δ* strain (Figure S4B, lanes 10–13), further supporting a link between 5′ and 3′ end maturation of 5.8S rRNA.

The protein Rai1p has previously been shown to function as a cofactor to Rat1p, and deletion of *RAI1* is synthetically lethal with the *rat1-1* mutation (El Hage et al., 2008; Xue et al., 2000). We tested if deletion of *RAI1* in an Rrp17p-depleted background had any further effect on 5.8S and 25S rRNA maturation. Removal of Rai1p exacerbated the effects of Rrp17p depletion only mildly (Figure 3Dc, lane 8). In comparison, deletion of *RAI1* in *P_MET_::rat1/xrn1Δ* cells had a much stronger effect on 5.8S maturation, resulting in one 5′-extended form and a shift to 5.8S_L_ rRNA (Figure 3Dc, lanes 8–10) (El Hage et al., 2008; Xue et al., 2000). A *P_GAL_::rrp17/P_MET_::rat1/xrn1Δ/rai1Δ* strain was not viable. Taken together, this indicates an essential role for Rrp17p in the 5′ exonucleolytic maturation of 5.8S_S_ and 25S rRNA, acting in parallel to the activity of Rat1p-Rai1p.

### Rrp17p Exhibits 5′–3′ Exonuclease Activity In Vitro

Rrp17p is a small (28 kDa) protein with a high proportion of the positively charged residues lysine (15.3%) and arginine (8.1%). Both amino acids are known to be present in many RNA-binding interfaces, as they enable RNA binding through interaction with the negatively charged phosphate backbone of RNA (Terribilini et al., 2006). We therefore tested whether Rrp17p exhibits an RNA-binding activity in vitro.

**Figure 4:**
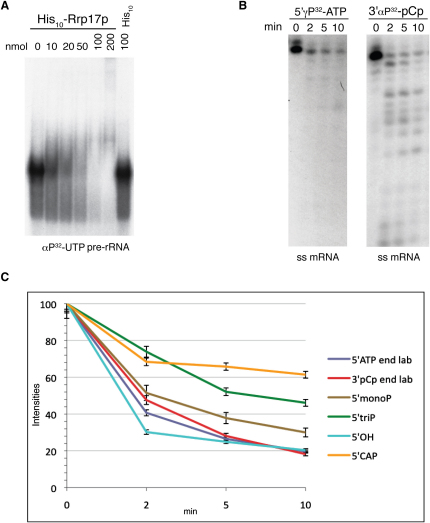
Analysis of RNA Binding and Exonuclease Function of Rrp17p In Vitro (A) Rrp17p binds to pre-rRNA in vitro. Gel mobility shift assay performed with an in vitro transcribed αP^32^-UTP-labeled pre-rRNA fragment and recombinant (His)_10_-Rrp17p. Lanes 1–6, pre-rRNA was incubated with 0–200 nmol Rrp17p as indicated; lane 7, pre-rRNA was incubated with 100 nmol (His)_10_. Complexes were resolved by electrophoresis in native 6% acrylamide/bisacrylamide. (B) Degradation of in vitro transcribed 5′γP^32^-ATP or 3′αP^32^-pCp end-labeled mRNA by Rrp17p. Nucleic acids were resolved on a 20% acrylamide/urea gels. For the times indicated, 0.5 pmol RNA was incubated with 50 nM Rrp17p at RT. (C) Degradation of RNA substrates containing different 5′ end modification by Rrp17p. The results of four experiments were averaged for each substrate and plotted against each other to determine degradation efficiency of Rrp17p in the presence of 5′ end modifications (error bars ±3%).

A gel shift assay was performed using a uniformly labeled pre-rRNA transcript, which extends from the 5′ region of ITS1 to the 3′ region of ITS2. Rrp17p clearly retarded the migration of the RNA, although the bound RNA did not migrate as a discrete species and appeared to degrade at higher concentrations of Rrp17p (as expected for a nuclease) (Figure 4A). However, in vitro binding of Rrp17p to RNA was not specific for pre-rRNA, as the protein bound equally well to in vitro transcribed pBluescript mRNA (data not shown).

In cells lacking both Rat1p and Xrn1p, the 5′ end of 5.8S_S_ rRNA is still synthesized, albeit with less efficiency, pointing toward the involvement of another, unknown nuclease (Figure 3C) (El Hage et al., 2008). We therefore determined whether Rrp17p exhibited nuclease activity in vitro. We examined degradation of an in vitro transcribed single-strand RNA labeled at its 5′ or 3′ end and incubated with recombinant Rrp17p. Incubation of protein with 5′ end-labeled RNA resulted in the loss of substrate without any detectable shortened fragments (Figure 4B, left); incubation of Rrp17p with 3′ end-labeled substrate resulted in the rapid and progressive degradation of the RNA (Figure 4B, right), revealing a 5′–3′ exonuclease activity of Rrp17p in vitro.

Rat1p and Xrn1p have previously been shown to be more active on substrates with a 5′ monophosphate than substrates containing a 5′ triphosphate (Poole and Stevens, 1997). To determine whether Rp17p was sensitive to the phosphorylation state of the 5′ end, we prepared short, uniformly labeled mRNA substrates that were either monophosphorylated or triphosphorylated at their 5′ ends and incubated with Rrp17p. Significant differences in the efficiency of degradation between 5′-monophosphorylated and triphosphorylated substrate (Figures 4C, S5A, and S5B) were observed, indicating that Rrp17p is indeed sensitive to the phosphorylation state of the 5′ end. We performed a similar analysis with substrates carrying a 5′-hydroxyl group or a 5′mG-cap structure, features known to inhibit Rat1p and Xrn1p activity (Poole and Stevens, 1997). However, while the 5′mG-cap containing substrate was less efficiently degraded than the control 5′ or 3′ end-labeled substrates, the 5′-hydroxyl end substrate was not (Figures 4C, S5C, and S5D); it therefore appears that Rrp17p has a somewhat different mode of function to Rat1p and Xrn1p. Lastly, we asked if Rrp17p has a specific requirement for divalent cations, as do many other nucleases. Using short, uniformly labeled mRNA substrates, the activity of Rrp17p was inhibited by replacement of MgCl_2_ with MnCl_2_ or by pretreatment with EDTA (Figures S5E and S5F). These results show that Rrp17p is a yeast exonuclease with Mg^2+^-dependent 5′–3′ activity in vitro and in vivo, which is required for 5′ end processing of 5.8S rRNA (Figures 3B and 3C, lanes 2–5).

### Rrp17p Possesses a Highly Conserved Domain that Is Important for Its Catalytic Activity

**Figure 5:**
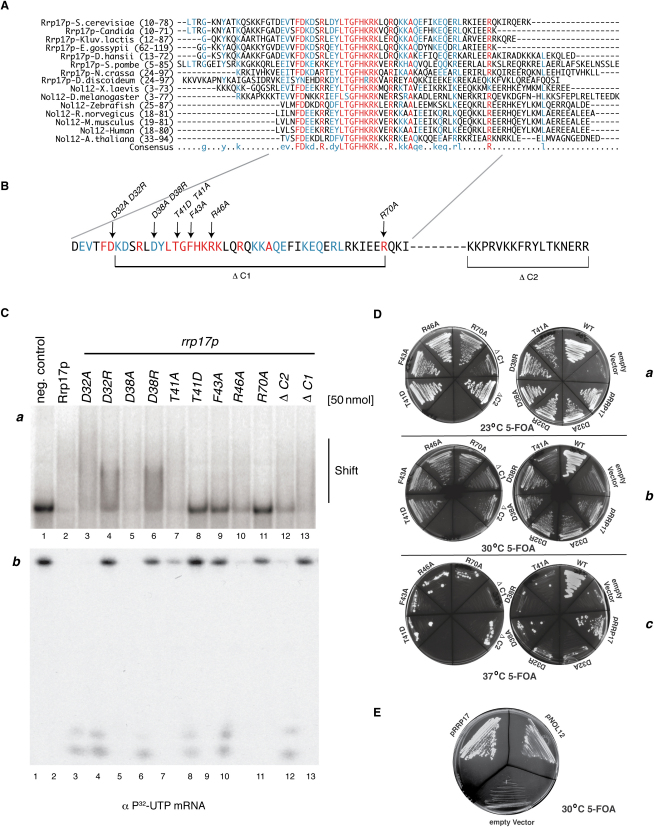
Rrp17p Contains a Highly Conserved Domain that Is Responsible for Exonuclease Activity (A) Alignment of Rrp17p with fungal and higher eukaryotic homologs using a ClustalW alignment algorithm (Altschul et al., 1997). Individual residues with more than 80% identity across the whole alignment are shown in red and as capital letters on the consensus line. Numbers in parentheses indicate the residue numbers of aligned sequences. (B) Schematic overview of point mutations introduced in the conserved domain of Rrp17p. (C) Top: Gel mobility shift assay performed with an in vitro transcribed pre-rRNA fragment. αP^32^-UTP pre-rRNA was incubated alone (lane 1) or with 50 nmol Rrp17p WT and mutant proteins for 30 min. Bottom: Degradation of in vitro transcribed 5′γP^32^-ATP mRNA by Rrp17p. RNA was incubated with 50 nM of either Rrp17p or mutant proteins for 10 min at RT. (D) As a control, an *RRP17* shuffle strain (*rrp17::KanMX6*/pURA3-*RRP17*) was transformed with an empty *TRP1* vector (pRS414). Phenotypes of *RRP17* point and truncation mutants were selected against by plating on 5-FOA plates, which causes the subsequent loss of pURA3-*RRP17*. Growth phenotypes were determined after growing transformants at 23°C, 30°C, and 37°C for 4 days. (E) Complementation of the *RRP17* deletion by wild-type *RRP17* and *NOL12* (h*RRP17*) was assessed by transforming the *RRP17* shuffle strain with constructs carrying wild-type *RRP17* or *NOL12*. The transformants were grown for 4 days at 23°C on medium containing 5-FOA.

Rrp17p does not share any significant sequence homology with any known exonucleases. However, database searches using PSI BLAST, ClustalW (UniProtK), and RNABindR revealed conserved Rrp17p homologs among fungi, protozoa, and higher eukaryotes, including *Dictystelium*, *Xenopus*, *Drosophila*, Zebrafish, *Arabidopsis*, mouse, and humans, with sequence similarities around 38% (Figure 5A) (Altschul et al., 1997; Terribilini et al., 2006). The human homolog Nol12, like Rrp17p, localizes mainly to the nucleolus (Fujiwara et al., 2006; Suzuki et al., 2006). All identified Rrp17p homologs contained a region of significant sequence similarity close to the N terminus; in yeast, this domain is found between amino acids 28 and 70 and is highly enriched in negatively and positively charged residues (Figure 5A). In addition, although not conserved on a sequence level, all homologs possessed a negatively charged region close to the C terminus, which was predicted to contain RNA-binding residues (RNABindR) (Terribilini et al., 2006).

To determine if the conserved domain is important for RNA binding or exonuclease activity of Rrp17p, we constructed nine point mutants targeting residues potentially involved in either function within the domain. The positively charged residues arginine, lysine, and histidine and the single aromatic residues phenylalanine and tyrosine all play key roles in RNA binding (Jones et al., 2001). Therefore, residues F43, R46, and R70 were each changed to alanine (Figure 5B). Aspartic acid residues have been found in the catalytic centers of different exonucleases; thus, we also changed the aspartic acid residues at sites 32 and 38 to alanine, as well as to arginine, as this has been shown to preserve RNA binding while disrupting any potential exonuclease activity (Terribilini et al., 2006). T41 is a residue that is entirely conserved in all homologs of Rrp17p, except *Drosophila*, where it has been replaced by serine. Thus, we converted T41 to an alanine residue. As both threonine and serine are kinase targets, this position represents a potential phosphorylation site (Figure 5A) (Terribilini et al., 2006), and so we also replaced T41 with aspartic acid residue, which mimics the negative charge state of a phosphorylated molecule (Egelhoff et al., 1993). We also constructed two truncation mutants, one lacking the entire conserved domain (*ΔC1*) and a second one lacking the less conserved, charged region toward the C terminus (*ΔC2*).

Gel shift and exonuclease assays were performed using uniformly labeled in vitro transcribed mRNA and bacterially expressed versions of each mutant (Figures S6B and S6C). RNA binding was not affected in *D32A* or *D38A* mutants, and these residues are thus unlikely to facilitate binding to the RNA (Figure 5Ca, lanes 3 and 5). Interestingly, while exonuclease activity was also not disrupted in the alanine conversion mutants (Figure 5Cb, lanes 3 and 5), both aspartic acid to arginine conversions (*D32R* or *D38R*) resulted in a loss of exonucleolytic activity (Figure 5Cb, lanes 4 and 6), while RNA binding was still observed (Figure 5Ca, lanes 4 and 6). The loss of activity could be due to disruption of charge-based interactions between the aspartic acid and nucleobases. An interesting effect was observed in mutants of T41; exonuclease activity, but not RNA binding, was reduced after a conversion to alanine (*T41A*) (Figures 5Cb, lane 7, and 5Ca, lane 7). Conversion to aspartic acid (*T41D*), however, mimicking a potential phosphorylated molecule, abolished both exonuclease activity and RNA binding (Figures 5Ca and 5Cb, lane 8), suggesting that T41 is indeed important for the catalytic activity of Rrp17p and might be regulated by phosphorylation. Strong inhibition of both RNA binding and exonuclease activity was also observed in *F43A* and *R70A* mutants (Figures 5Ca and 5Cb, lanes 9 and 11), but not *R46A* (Figured 5Ca and 5Cb, lane 10), indicating that residues 43 and 70 are most likely part of the catalytic and RNA-binding domain. The removal of the conserved domain (*ΔC1*) had only a minor effect on RNA-binding efficiency (Figure 5Ca, lane 13), but no exonuclease activity was observed in these mutants; deletion of the C-terminal domain (*ΔC2*), however, did not affect catalytic activity but decreased RNA binding to the substrate (Figures 5Ca and 5Cb, lane 12). Thus, both C1 and C2 contain RNA-binding activity, but only C1 contains catalytic activity.

To define the importance of the conserved C1 domain in cell growth, the mutated *rrp17* ORFs were tested for their ability to rescue an *RRP17* deletion (Figure 5Da). All the mutant proteins were stably expressed (Figures S6D and S6E), and all alleles, except *ΔC1*, were viable at 23°C (Figure 5Da). However, viability of the mutants, but not wild-type or a strain expressing wild-type RRP17, was greatly reduced at 37°C (Figure 5Dc), while *T41A* and *R70A* already exhibited reduced viability at 30°C (Figure 5Db). These data support our in vitro analysis of Rrp17p mutants; moreover, the strong phenotype associated with the T41 mutant, deficient in catalytic activity but not RNA binding, emphasizes the likely importance of Rrp17p exonuclease activity in vivo.

### Deletion of Rrp17p Is Rescued by the Human Rrp17p Homolog Nol12

The high sequence conservation observed between Rrp17p homologs suggested that its function might be conserved to higher eukaryotes. To test this, the coding sequence of the human homolog NOL12 was amplified from spleen cDNA, cloned into a yeast expression vector, and tested for its ability to rescue viability in the *rrp17Δ* strain. The Nol12 construct supported viability (Figure 5E), establishing that it is a conserved, functional homolog of yeast Rrp17p.

### Functional Links between Late Processing and Export of Pre-60S Ribosomal Subunits

**Figure 6:**
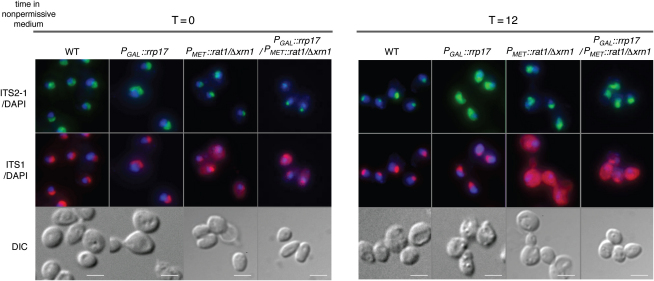
Export of Pre-60S Subunits Is Affected by Depletion of both Rrp17p and Rat1p Localization of pre-60S (ITS2-1) and pre-40S (ITS1) ribosomal subunits in wild-type, *P_GAL_::rrp17*, *P_MET_::rat1/xrn1Δ*, and *P_GAL_::rrp17/P_MET_::rat1/xrn1Δ* cells. Cells were grown in permissive medium to mid log phase and then shifted to restrictive medium for up to 12 hr before being fixed and mounted with DAPI (blue) to stain the nuclei. Nucleolar distribution of 5′ITS2-1 (green) and ITS1 (red) was determined in permissive and restrictive conditions. Bars represent 10 μm.

Rrp17p-associated complexes not only contained pre-60S subunit components, but also NPC components and proteins known to chaperone preribosomes through the NPC into the cytoplasm. We therefore assessed whether export of preribosomes was affected by depletion of Rrp17p. The localization of pre-60S subunits was determined by fluorescence in situ hybridization (FISH) using a probe complementary to the 5′ end of ITS2 (a region present in 35S, 32S, 27S, and 7S pre-rRNAs) (Léger-Silvestre et al., 2004; Zenklusen et al., 2008). In both the wild-type and the *P_GAL_::rrp17* cells grown under permissive conditions (T = 0), the ITS2-containing pre-rRNAs were concentrated in the nucleolus (Figure 6). Following growth in glucose medium for 12 hr, in >90% of cells, the signal was found to accumulate throughout the nucleoplasm in Rrp17-depleted but not wild-type cells (Figure 6), where it was still localized to the nucleolus. The localization of pre-40S subunits was determined in the same cells by using a probe complementary to the 5′ end of ITS1 (Moy and Silver, 1999). No difference in localization of ITS1 signal was seen in Rrp17p-depleted cells compared to wild-type after 12 hr in glucose (Figure 6). Hence, depletion of Rrp17p hinders the export of 60S ribosomal subunits, but not 40S subunits.

To further investigate a potential link between late processing of rRNA and ribosomal export, we also assessed export of preribosomal subunits in the absence of Rat1p and Xrn1p. In *P_MET_::rat1/xrn1Δ* cells grown under permissive conditions (T = 0), the ITS2-containing pre-rRNAs were concentrated in the nucleolus (Figure 6). However, similar to Rrp17p-depleted cells, following growth under restrictive conditions for 12 hr, the ITS2 signal was found to accumulate throughout the nucleoplasm, although to a much lesser extent (>85%) (Figure 6). No nuclear accumulation of ITS1 signal was seen in *P_MET_::rat1/xrn1Δ* cells, indicating that pre-40S export was not affected in those cells (Figure 6). The deletion of Xrn1p, however, resulted in an accumulation of excised D-A_2_ RNA in the cytoplasm, as the protein is responsible for degradation of this fragment (Figure S7A) (Moy and Silver, 1999). Export of pre-60S ribosomal subunits was also disrupted in *P_GAL_::rrp17/P_MET_::rat1/xrn1Δ* (Figure 6) and *P_GAL_::rrp17/P_MET_::rat1* cells (>90%) (Figure S7B). Taken together, these data show that defects in 5′ maturation of 5.8S and 25S rRNAs hinder pre-60S subunit export and, moreover, point toward a link between late processing of rRNA and ribosomal export. To determine whether this phenomenon is specifically true for 5′ maturation of pre-60S components or extends to late maturation steps in general, localization of ITS2-containing pre-rRNAs was determined in the absence of Rai1p, the cofactor to Rat1p, and Rrp6p, a 3′–5′ exonuclease required for 3′ maturation of 5.8S rRNA (Briggs et al., 1998) (Figure S7C). ITS2 was found to accumulate throughout the nucleoplasm in the absence of either protein (>90%), while localization of ITS1 and thus export of pre-40S subunits did not seem affected (Figure S7C). This points strongly to a coupling between both late 5′ and 3′ maturation events of pre-60S RNA components and pre-60S subunit export.

**Figure 7:**
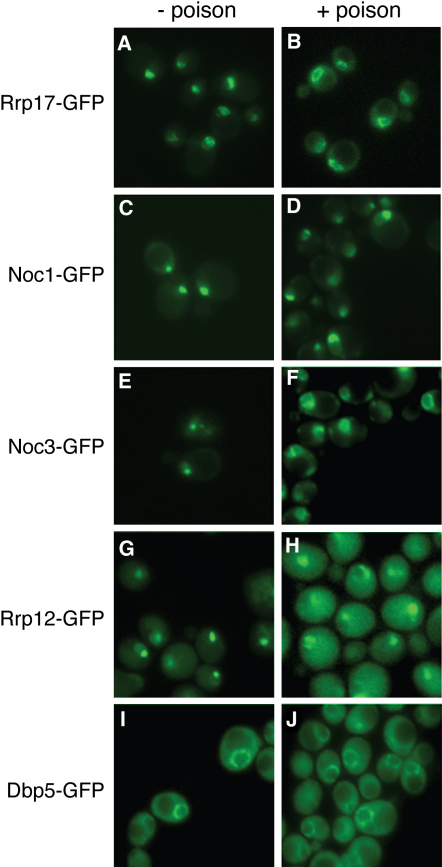
Conditions that Disrupt Nuclear Transport Lead to a Redistribution of Late Preribosomal Processing Factors to the Nuclear Periphery (A–J) Nuclear transport was disrupted using a metabolic poison cocktail (Shulga et al., 1996). Localization of GFP-tagged Rrp17p (A and B), Noc1p (C and D), Noc3p (E and F), Rrp12p (G and H), and Dbp5p (I and J) was determined in vivo prior to poison treatment and after 30 min in metabolic poison. Bars represent 10 μm.

If indeed there is a direct functional link between late processing and nuclear export of pre-60S ribosomal subunits, then one might expect that disruption of nuclear transport might cause a redistribution of proteins specifically involved in late 60S processing, of which Rrp17p would be a prominent example. Indeed, we find such a redistribution. Both Rrp17p and another late processing factor, Noc3p (Milkereit et al., 2001), redistribute (in >95% of cells) upon exposure to a metabolic poison cocktail that is known to reversibly arrest nuclear transport (Figures 7A, 7B, 7E, and 7F) (Shulga et al., 1996). Interestingly, both proteins accumulate at the nuclear periphery, consistent with their biochemical association with the NPC and nuclear peripheral components (Figure 1A). By contrast, the early ribosomal processing factor Noc1p (Milkereit et al., 2001), did not redistribute under these conditions (Figures 7C and 7D), underscoring the specificity of this result. It seems unlikely that Rrp17p is involved in the transport of ribosomes, as its redistribution in poison resembles that of Noc3p but not of Rrp12p (Oeffinger et al., 2004), a shuttling factor known to facilitate ribosome export, which becomes more cytoplasmic upon metabolic arrest (Figures 7G and 7H). The control protein Dbp5p (Strahm et al., 1999), an mRNA helicase localized to the NPC and cytoplasm, does not redistribute in these conditions (Figures 7I and 7J).

## Discussion

We show here that Rrp17p is a previously unknown 5′–3′ exonuclease, which we identified in proteomic studies of preribosomal complexes where it is present in intermediate to late pre-60S subunits and associates with precursors to 5.8S and 25S rRNAs. Rrp17p is an essential protein that binds late pre-60S ribosomes and is required for efficient exonuclease digestion to the mature 5′ ends of 5.8S_S_ and 25S RNAs. Moreover, it accompanies the 60S subunit from the nucleolus to the nuclear periphery and even to the NPC. With a putative catalytic domain close to its N terminus, Rp17p is highly conserved among fungi and higher eukaryotes, though it lacks homology to other known nucleases. The human homolog, Nol12, was able to replace Rrp17p in vivo, confirming the functional conservation of this protein family.

### Rrp17p Is Required for the Efficient Formation of 5.8S_S_ rRNA and 25S rRNA

The 5′–3′ exonuclease Rat1p was previously implicated as the key player in the 5′ maturation of rRNAs in yeast. However, Rat1p has a homolog, Xrn1p; in the absence of Rat1p, it has been suggested that Xrn1p substitutes for Rat1p's function (El Hage et al., 2008; Geerlings et al., 2000; Johnson, 1997). The characterization of Rrp17p reveals that, similar to 3′ end processing of 5.8S rRNA, 5′ end maturation also involves multiple nucleases. The presence of another nuclear 5′–3′ exonuclease provides the cell with redundant pathways to ensure robust and efficient production of 60S ribosomal subunits. Interestingly, using in vitro transcribed pre-RNA substrates starting from either cleavage sites at A_3_ or C_2_, degradation did not occur for the same number of nucleotides for the two substrates (Figures S7G and S7H). Instead, processing was stopped at different distances from the 5′ end in each case, suggesting that the enzyme is most likely stopped by either secondary structure or nonpreferred sequence. The latter is known to exist for Rat1p, which is inhibited by poly-G-rich sequences (Poole and Stevens, 1997). This suggests that, although able to degrade ITS regions on their own, each exonuclease is likely to have preferred substrate regions within ITS1 and 2, underlining the potential gain in efficiency provided by another exonuclease and a shared role during 5′ end pre-rRNA maturation. We therefore speculate that both Rrp17p and Rat1p associate with each pre-rRNA substrate during processing to orchestrate the maturation process.

Rai1p, the *Ra*t1p-*i*nteracting protein, has been reported to enhance the activity of Rat1p (Xue et al., 2000). Deletion of *RAI1* leads to a discernable shift in the 5.8S_S_ to 5.8S_L_ ratio, which is complete in the absence of Rat1p, an effect that is not seen in *rrp17/rai1* mutants, where deletion of Rai1p results only in a mild exacerbation of the 5′ end processing defect. Moreover, a direct interaction was shown for Rat1p and Rai1p in vitro, and Rai1p relocalizes to the cytoplasm in *rat1* mutants, making it unlikely that Rai1p also functions as a cofactor for Rrp17p (Sydorskyy et al., 2003). However, the association of Rai1p with pre-rRNA may be required for conformational changes within ITS1 that allow efficient exonucleolytic processing by both nucleases, which could explain the increase in endonucleolytic processing and shift in 5.8S_S_ to 5.8S_L_ ratio in *rai1Δ* cells.

Very little is known about whether processing within the two ITS regions is coordinated; however, it has been observed that defective processing in ITS1 can lead to either premature or less-efficient processing in ITS2 (Côté et al., 2002; Eppens et al., 2002; Fatica et al., 2002; Shuai and Warner, 1991). In the absence of an efficient exonucleolytic 5′ end processing pathway, a gradual shift to endonucleolytic 5′ end maturation of 5.8S rRNA and increase in 5.8S_L_ occurs. In agreement with previous studies, the concomitant accumulation of ITS2-containing precursors (27SB, 26S, and 7S) suggests a linkage between the processing of both spacers. Specifically, the appearance of 3′-extended 5.8S precursor RNAs in both Rrp17p- and Rat1p-depleted strains implies a connection between 5′ and 3′ end maturation of 5.8S rRNA (El Hage et al., 2008). Indeed, in the past, it has been suggested that Rai1p may coordinate the 5′ end and 3′ end processing activities of Rat1p and the nuclear exosome, as deletion of *RAI1* strongly accumulated 3′-extended precursors of 5.8S rRNA and was synthetically lethal when combined with a deletion of the exosome component *RRP6* (El Hage et al., 2008; Fang et al., 2005).

### The Association of Rrp17p with the Nuclear Periphery

Biochemical, functional, and localization evidence all agree that Rrp17p can be associated with the nuclear periphery and the NPC. Upon immunoisolation, 13 nucleoporins, nuclear peripheral proteins (such as the Mlp proteins), and nuclear transport factors copurify with tagged Rrp17p. This provides strong evidence for a close physical connection between Rrp17p and regions on and immediately surrounding the NPC's nuclear face. We also show that Rrp17p partially colocalizes with the nuclear periphery in vivo and that this localization is significantly and specifically enhanced by conditions that disrupt nuclear transport. A functional connection between Rrp17p's role in late ribosomal processing and nuclear export is shown by the inhibition of late pre-60S export upon depletion of Rrp17p. It is formally possible that the pool of Rrp17p associated with the NPC is not the same as the pool involved in late 60S processing; however, taken together, the most parsimonious interpretation of our data is that Rrp17p accompanies the late 60S subunit to the nuclear periphery and NPC and that correct processing is a prerequisite for export of the subunit through the NPC. Indeed, we provide evidence that there may be a general physical and functional link between late processing of 60S subunits and export, as the latter is not only delayed in Rrp17p-depleted cells but also in strains carrying mutants of Rat1p, Xrn1p, Rai1p, or Rrp6p—all components of the late 5′ and 3′ processing machinery. Such a link would not be altogether surprising, as a similar mechanism has been identified for the late mRNA processing and proofreading machinery, which associates with the nuclear periphery and NPC (Carmody and Wente, 2009).

### Rrp17p Is Part of a Family of Conserved Eukaryotic Exonucleases

Rrp17p is just one member of a conserved family of putative RNA exonucleases (Figure 7). Although the rat homolog of Rrp17p, Nop25, has previously been characterized in COS7 cells as a nucleolar RNA-binding protein involved in ribosome synthesis, no functional data were shown at the time (Fujiwara et al., 2006; Suzuki et al., 2006). The ability of Nol12 to rescue a *RRP17* deletion, however, denotes the protein not only as a sequence but also as a functional homolog of Rrp17p, and it will be interesting to determine if Nol12 fulfills a similar role during rRNA processing in higher eukaryotes to Rrp17p. So far, we are only slowly unraveling the mechanism of ribosome maturation in higher eukaryotes, due to its even greater complexity. The characterization of Nol12 will therefore provide us with useful insight into ribosome biogenesis in mammalian cells. Moreover, given the multifunctional nature of Xrn2/Rat1p as an exonuclease in mRNA degradation as well as rRNA processing (Kim et al., 2006), it will be interesting to see if the sole function of Nol12 and Rrp17p lies in rRNA maturation or if the protein has additional roles in degradation of other RNA species.

## Experimental Procedures

### Yeast Strains

Growth and handling of *S. cerevisiae* were carried out using standard techniques. PrA-tagged strains were generated in the wild-type strain W303 as described (Rout et al., 2000). Conditional mutants under the control of repressible GAL10 and MET3 promoters, as well as epitope-tagged and deletion strains, were generated by a one-step PCR strategy in the wild-type W303 strain (Longtine et al., 1998). Transformants were selected for G418 resistance and screened by PCR. The *P_Met3_-Rat1::HIS3/xrn1Δ::NAT, rai1Δ::KANMx6* and *P_Met3_-Rat1::/xrn1Δ::NAT/rai1Δ::KANMx6* were kindly provided by A.E.H. and D.T. (El Hage et al., 2008). The shuffle strain was constructed as described in the Supplemental Experimental Procedures. The yeast strains used in this work are listed in Table S1; plasmids are listed in Table S2.

### Immunoaffinity Purification

Cells were harvested by centrifugation, and the frozen cells were ground in a Planetary Ball Mill (PM 100; Retsch; Newtown, PA) using 20 mm stainless-steel bearings. Immunoaffinity purification and mass spectrometric analysis of Rrp17-associated complexes were carried out as described in Oeffinger et al., 2007 and the Supplemental Experimental Procedures.

### RNA Extraction, Northern Hybridization, and Primer Extension

RNA extractions, northern hybridizations, and primer extension analysis were carried out as described in the Supplemental Experimental Procedures. Standard 1.2% agarose/glyoxal and 6% acrylamide/urea gels were used to analyze the high- and low-molecular-weight RNA species, respectively. Oligos used for RNA hybridizations are listed in the Supplemental Experimental Procedures.

### Western Blots

Total protein extracts and western blot analysis were performed as previously described (Oeffinger et al., 2007) (Supplemental Experimental Procedures).

### Recombinant Protein Purification

A PCR fragment corresponding to the Ydl412w (RRP17) ORF was amplified and cloned into pKS132-His_10_ vector (L. Westerblade and S. Darst) using NdeI-NotI. (HIS)_10_-Rrp17p and all RRP17 mutants were expressed in *E. coli* strain BL21(DE3)RIL at 30°C for 4 hr. The proteins were purified in buffer A (50 mM Tris [pH 7.5], 200 mM NaCl, 5 mM MgCl_2_, 80 mM imidazol) using magnetic nickel resin (Ademtech; Pessac, France) according to the manufacturer's protocol. The protein was dialyzed against 50 mM Tris (pH 7.5), 50 mM NaCl, and 5 mM MgCl_2_.

### Cosedimentation and Velocity Gradient Analysis

Sucrose gradient centrifugation was performed as described in the Supplemental Experimental Procedures. RNA was extracted from each fraction and resolved on standard 1.2% agarose/formaldehyde gel. Mature rRNAs and pre-rRNA species were detected by ethidium staining and northern hybridization, respectively. Sedimentation of proteins was assayed by SDS-PAGE, and PrA-tagged Rrp17p was detected by western immunoblotting with peroxidase-conjugated rabbit IgG (Sigma; St. Louis). Velocity centrifugations were carried out on a 5%–20% (w/w) sucrose gradient as described in Alber et al., 2007.

### RNA Mobility Shift and Exonuclease Assays

Labeled and unlabeled RNA substrates were synthesized in vitro by T7 polymerase transcription of linearized pBluescript and of rDNA (5′-ITS1 to 3′-ITS2) that had been amplified from an rDNA plasmid and is carrying the T7 promoter region. Band-shift and exonuclease assays were performed as described in the Supplemental Experimental Procedures.

### Fluorescent Microscopy, In Situ Hybridization, and Cell Imaging

For FISH, cells growing in permissive or shifted to nonpermissive medium for 12 hr were fixed and hybridized with pre-rRNA probes as described in Zenklusen et al., 2008 and Supplemental Experimental Procedures. Images were acquired using an Olympus BX61 wide-field epifluorescence microscope using an Olympus 100×, 1.35NA objective with HC DIC.

### Poison Assay

GFP-tagged strains were grown to early/mid log phase for steady-state analysis and then treated essentially as described by Shulga et al., 1996, using metabolic energy poisons to arrest ATP-dependent steps. A detailed description of the methods used is available in the Supplemental Experimental Procedures.

## Supplemental Data

Document S1. Supplemental Experimental Procedures, Supplemental References, Seven Figures, and Two Tables
