# Supplementary material for: Rrp17p Is a Eukaryotic Exonuclease Required for 5′ End Processing of Pre-60S Ribosomal RNA
Source: Mol Cell. 2009 Dec 11;36(5):768–81. doi: 10.1016/j.molcel.2009.11.011 (PMC2806520; doi:10.1016/j.molcel.2009.11.011)
Supplement: Document S1. Supplemental Experimental Procedures, Supplemental References, Seven Figures, and Two Tables [file mmc1.pdf]

Supplemental Data
Molecular Cell, *Volume 36*

Rrp17p Is a Eukaryotic Exonuclease Required for 5′ End Processing of Pre-60S Ribosomal RNA

Marlene Oeffinger, Daniel Zenklusen, Angelica Ferguson, Karen E. Wei, Aziz El Hage, David Tollervey, Brian T. Chait, Robert H. Singer, and Michael P. Rout

SUPPLEMENTAL EXPERIMENTAL PROCEDURES

Yeast Strains

ProteinA (PrA)-tagged strains were generated in the wild-type strain W303 as described (Rout et al., 2000). Conditional mutants under the control of repressible GAL10 and MET3 promoters were generated by a one-step PCR strategy in W303 (Longtine et al., 1998). Transformants were selected for G418 resistance and screened by PCR. The *P_{Met3}-Rat1::HIS3/xrn1Δ::NAT, rai1Δ::KANMx6* and *P_{Met3}-Rat1::/xrn1Δ::NAT/rai1Δ::KANMx6* were kindly provided by A. El Hage and D. Tollervey (El Hage et al., 2008). HA-tagging of *P_{Met3}-Rat1::HIS3/xrn1Δ::NAT* was carried out by a one-step PCR strategy, inserting a 3xHA cassette. Deletion of RAI1 in the KanMX6-Gal::HA_3-*rrp17* was carried out by replacing it with a URA3 cassette. The shuffle strain was constructed as follows; first, RRP17 including 5' and 3' flanking sequences (500 bp) were amplified as *Bam*HI-*NotI* fragment and cloned into pRS416-URA3. The plasmid was transformed into a diploid W303 strain, in which one allele of RRP17 had been replaced by *KanMX6* cassette (Longtine et al., 1998). Cells were sporulated, tetrads dissected, and haploid cells selected to for both, URA^+ autotroxophy and G418 resistance. Point and truncation mutants of RRP17 were constructed by Mutagenex Inc. in pKS132-His_{10}, and cloned into pRS414-HA-TRP1, containing the RRP17 promoter region and terminator of the PGK1 gene, using *NdeI-NotI.* Human RRP17 (Nol12) was amplified from human spleen cDNA and cloned into pRS414-HA-TRP1 using *NdeI-NotI.* Strains expressing RRP17, *rrp17* mutants or NOL12 were obtained by transformation of the RRP17 shuffle strain with wildtype, mutant pRS414-3xHA-RRP17 or pRS414-

3xHA-NOL12 plasmid followed by selection against the pURA3-RRP17 plasmids on 5-fluororotic acid (5-FOA) plates. The Yeast strains used in this work are listed in Table S1; plasmids are listed in Table S2.

Growth and handling of *S.cerevisiae* were by standard techniques. PrA-tagged strains were grown to mid-log phase (OD_{600} ~0.8) in medium containing 2% glucose. GAL-regulated strains were pre-grown in RGS or synthetic complete (SC) medium, containing 2%raffinose, 2% galactose and 2% sucrose, at 30°C to OD_{600} ~0.4, and transferred to full or SC medium containing 2% glucose. For Rat1p depletion, cells were grown at 30°C to OD_{600} ~0.4 in SD medium lacking methionine, then were transferred to the same prewarmed medium supplemented with 5mM methionine. The growth for both strains was continued for several hr and maintained in exponential phase by dilution with prewarmed medium. 10 cell ODs were harvested at 2-3 hr intervals.

Immuno-Affinity Purification

Cells were harvested by centrifugation and the frozen cells ground in a Planetary Ball Mill (PM 100; Retsch) using 20mm stainless-steel bearings as described (Oeffinger et al., 2007). Cell grindates were stored at -80°C until used.

2.5g of frozen cell grindate was thawed into RNPB buffer (20mM Hepes, pH7.5, 110mM KOAc, 0.5% Triton X-100, 0.1% Tween20, 1:200 Solution P, 1:1000 SuperRNasin, 1:5000 Antifoam B). The cell lysates were clarified by filtration. Rrp17-PrA associated complexes were isolated using IgG-conjugated magnetic beads (Dynal) as previously described (Oeffinger et al., 2007). The isolated protein complex was eluted from the beads in fresh aqueous 500 mM NH_4OH, 0.5 mM EDTA solution. The pooled eluates were lyophilized in a SpeedVac (Thermo Savant) overnight. The pellets were resuspended in SDS-PAGE sample buffer, separated on a 4–12% NuPAGE Novex Bis-Tris precast gel (Invitrogen) and visualized by R250 Coomassie blue staining. Gel bands were excised in 1-mm

sections, treated as previously described (Oeffinger et al., 2007). A MALDI orthogonal time of flight (prOTOF 2000, Perkin Elmer Science) and a MALDI linear ion trap mass spectrometer (vMALDI LTQ, Thermo Fisher Scientific) were used for peptide fingerprinting (MS) and amino acid sequencing (MS/MS), respectively (Krutchinsky, 2001). XProteo (www.xproteo.com) was used to correlate peptide mass fingerprint data or tandem MS CID data obtained from MS and MS/MS analyses and enabled identification of proteins (Ossipova et al., 2006).

RNA Extraction, Northern Hybridization, and Primer Extension

RNA was extracted as described previously (Kufel et al., 2000). Northern hybridizations and primer extension analysis were as described (Kufel et al., 2000; Thomson and Tollervey, 2005). Standard 1.2% agarose/glyoxal and 6% acrylamide/urea gels were used to analyze the high and low molecular weight RNA species, respectively. For RNA hybridizations, the following oligonucleotides were used:

001-complementary to ITS1 downstream of A_3, 5'-CCAGTTACGAAAATTCTTG;

003- complementary to ITS1 between A_2 and A_3, 5'-TGTTACCTCTGGGCCC;

004- complementary to 5'ITS downstream of D, CGGTTTTAATTGTCCTA;

005-complementary to ITS1 between A_2 and A_3, 5'-ATGAAAACTCCACAGTG;

006-complementary to the ITS2 region upstream of C_1, 5'-GGCCAGCAATTTCAAGTTA;

007-complementary to the 25S rRNA, 5'-CTCCGCTTATTGATATGC;

008-complementary to 18S rRNA, 5'-CATGGCTTAATCTTTGAGAC;

017-complementary to 5.8S rRNA, 5'-GCGTTGTTCATCGATGC;

020-complementary to the 5.8S/ITS2 boundary, 5'-TGAGAAGGAAATGACGCT;

033-complementary to 5'ETS 278nt downstream of A_0, 5'-CGCTGCTCACCAATGG; 041-complementary to 5S rRNA, 5'-CTACTCGGTCAGGCTC.

Western Blots

Total protein extracts and western blot analysis were performed as previously described (Oeffinger et al., 2007). Both *Gal::HA_3-rrp17* and *Met::HA_3-Rat1* were detected with mouse anti-HA sc-7392 antibody (Santa Cruz Biotechnology), PGK1 was detected with mouse anti-PGK1 antibody (Molecular Probes).

Recombinant Protein Purification

A PCR fragment corresponding to the Ydl412w (RRP17) ORF was amplified and cloned into pKS132-His_{10} vector (a kind gift of L. Westerblade and S. Darst) using *NdeI-NotI*. $(HIS)_{10}$-Rrp17p and all RRP17 mutants were expressed in *E.coli* strain BL21(DE3)RIL at 30°C for 4h. The proteins were purified in buffer A (50mM Tris pH7.5, 200mM NaCl, 5mM $MgCl_2$, 80mM imidazol) using magnetic nickel resin (Ademtech) according to the manufacturer's protocol. The protein was dialyzed against 50mM Tris pH7.5, 50mM NaCl and 5mM $MgCl_2$.

Cosedimentation and Velocity Gradient Analysis

Sucrose gradient centrifugation was performed as described (Baßler et al., 2001; Tollervey et al., 1993). RNA was extracted from each fraction and resolved on standard 1.2% agarose/formaldehyde gel. Mature rRNAs and pre-rRNA species were detected by ethidium staining and Northern hybridization, respectively. Sedimentation of proteins was assayed by SDS-PAGE and PrA-tagged Rrp17p was detected by Western immunoblotting with peroxidase-conjugated rabbit IgG (SIGMA). Velocity Centrifugations were carried out on a 5-20% (w/w) sucrose gradient as described in (Alber et al., 2007). The markers used were aprotinin, cytochrome C, carbonic anhydrase and bovine serum albumin (Sigma). Briefly, 5µg of recombinantly purified Rrp17p and marker proteins were centrifuged on a sucrose/TB (20mM Hepes, pH7.5, 110mM KOAc, 0.1% Tween 20, 1mM DTT, 1:200 Solution P) gradient at ~300,000 g_{max} for 24h in an SW55 Ti rotor at 4 °C. 200µl fractions were collected from the

top of the gradient and analyzed by SDS-PAGE and Coomassie staining. Band intensities were quantified using ImageJ, and the peak fractions of the marker proteins were plotted as function of its sedimentation coefficient and fitted with a standard curve by linear regression (r-squared >0.99 in all cases).

RNA Mobility Shift and Exonuclease Assays

Labeled and unlabeled RNA substrates were synthesized *in vitro* by T7 polymerase transcription of pBluescript(+) linearized with *XbaI*, and of rDNA (5'-ITS1 to 3'-ITS2) that had been amplified from an rDNA plasmid and is carrying the T7 promoter region. RNAs were purified on 15% acrylamide/urea gels, excised from the gel, eluted and precipitated. The RNA mobility shift binding reaction was performed in 30mM Tris-HCl pH 7.4, 150 mM KCl, 2mM $MgCl_2$, 0.1% Triton X-100, 20% glycerol and 1mM DTT, in the presence of tRNA (1mg/ml), 0.25pmol of ^{32}P-labelled pre-rRNA and 0-200nmol of recombinant protein in a reaction volume of 15 µl. RNA was heat denatured at 65°C for 10 min, followed by slow cooling to room temperature, and then added to the binding reaction. Reactions were incubated at room temperature for 30 min and then loaded on a 6% native acrylamide/bisacrylamide (80:1)/4% glycerol gel in 0.5x TBE buffer. Prior to loading, the gel was pre-run for 1 h and then run for 3h at 250 V in the cold room (Fatica et al., 2002b).

Exonuclease assays were performed in 10mM Tris-HCl pH 7.6, 50mM KCl, 1mM $MgCl_2$, 10mM DTT, 100µg/ml BSA and 0.8U/µl in the presence of 0.5pmol ^{32}P-labelled mRNA and 50mM of recombinant protein in 15µl total reaction volume. Samples were incubated at room temperature for 0-15min and then loaded onto 20% acrylamide/urea gels in 1x TBE buffer and run for 2hs at 200V.

Fluorescent Microscopy, In Situ Hybridization, and Cell Imaging

For fluorescent in situ hybridization (FISH), cells growing in permissive or shifted to non-permissive medium for 12hr were fixed and hybridized with pre-rRNA probes as described in (Zenklusen et al., 2008). ITS1 probe (TGGAC**T**CTCCATCTCTTGAC**T** TCTTGCCCAG**T**AAAAGCTC**T**CATGCTC**T**T) labeled with Cy5 and ITS2-1 probe (A**T**AGGCCAGCAATTTCAAGT**T**AACTCCAAAGAGTATCAC**T**C) labeled with Cy3 were used. Bold letters indicate labeled nucleotides.

All images were acquired using a Olympus BX61 wide-field epi-fluorescence microscope using an Olympus 100x, 1.35NA objective with HC DIC optics from cells grown in synthetic medium lacking histidine. Multiple fields of cells were counted (~300) and % of cells determined displaying phenotype.

Poison Assay

GFP-tagged strains were grown to early-mid-log phase (A600 = 0.4) for steady-state analysis and then treated essentially as described by (Shulga et al., 1996) using metabolic energy poisons to arrest ATP-dependent steps. 10 ml of cells were harvested at room temperature, washed with 1 ml ddH20; 500µl of washed cell suspension were pelleted again and resuspended in 1 ml of 10 mM sodium azide ("azide"), 10 mM 2-deoxy-o-glucose ("deoxyglucose") in glucosefree SC medium and incubated for 30 min at 30°C. Poison-treated cells and untreated cells were then imaged using a Olympus BX61 wide-field epi-fluorescence microscope using an Olympus 100x, 1.35NA objective.

SUPPLEMENTAL REFERENCES

Alber, F., Dokudovskaya, S., Veenhoff, L. M., Zhang, W., Kipper, J., Devos, D., Suprapto, A., Karni-Schmidt, O., Williams, R., Chait, B. T., *et al.* (2007). Determining the architectures of macromolecular assemblies. Nature *450*, 683-694.

Baßler, J., Grandi, P., Gadal, O., Leßmann, T., Petfalski, E., Tollervey, D., Lechner, J., and Hurt, E. (2001). Identification of a 60S pre-ribosomal particle that is closely linked to nuclear export. Mol Cell *8*, 517-529.

El Hage, A., Koper, M., Kufel, J., and Tollervey, D. (2008). Efficient termination of transcription by RNA polymerase I requires the 5' exonuclease Rat1 in yeast. Genes Dev *22*, 1069-1081.

Fatica, A., Dlakic, M., and Tollervey, D. (2002b). Naf1 p is a box H/ACA snoRNP assembly factor. RNA *8*, 1502-1514.

Krutchinsky, A. (2001). Automatic identification of proteins with a MALDI-quadrupole ion trap mass spectrometer. Anal Chem *73*, 5066-5077.

Kufel, J., Allmang, C., Chanfreau, G., Petfalski, E., Lafontaine, D., and Tollervey, D. (2000). Precursors to the U3 snoRNA lack snoRNP proteins but are stabilized by La binding. Mol Cell Biol *20*, 5415-5124.

Longtine, M., McKenzie, A., Demarini, D., Shah, N., Wach, A., Brachat, A., Philippsen, P., and Pringle, J. (1998). Additional modules for versatile and economical PCR-based gene deletion and modification in Saccharomyces cerevisiae. Yeast *14*, 953-961.

Oeffinger, M., Wei, K., Rogers, R., DeGrasse, J., Chait, B., Aitchison, J., and, and Rout, M. (2007). Comprehensive analysis of diverse ribonucleoprotein complexes. Nat Methods *Nov; 4:* , 951-956. Epub 2007 Oct 2007.

Ossipova, E., Fenyo, D., and Eriksson, J. (2006). Optimizing search conditions for the mass fingerprint-based identification of proteins. Proteomics *6*, 2079-2085.

Rout, M., Aitchison, J., Suprapto, A., Hjertaas, K., Zhao, Y., and Chait, B. (2000). The yeast nuclear pore complex: composition, architecture, and transport mechanism. J Cell Biol *148*, 635-651.

Shulga, N., Roberts, P., Gu, Z., Spitz, L., Tabb, M., Nomura, M., and Goldfarb, D. (1996). In vivo nuclear transport kinetics in Saccharomyces cerevisiae: a role for heat shock protein 70 during targeting and translocation. J Cell Biol *135*, 329-339.

Thomson, E., and Tollervey, D. (2005). Nop53p is required for late 60S ribosome subunit maturation and nuclear export in yeast. Rna *11*, 1215-1224.

Tollervey, D., Lehtonen, H., Jansen, R., Kern, H., and Hurt, E. (1993). Temperature-sensitive mutations demonstrate roles for yeast fibrillarin in pre-rRNA processing, pre-rRNA methylation, and ribosome assembly. Cell *72*, 443-457.

Zenklusen, D., Larson, D. R., and Singer, R. H. (2008). Single-RNA counting reveals alternative modes of gene expression in yeast. Nat Struct Mol Biol.

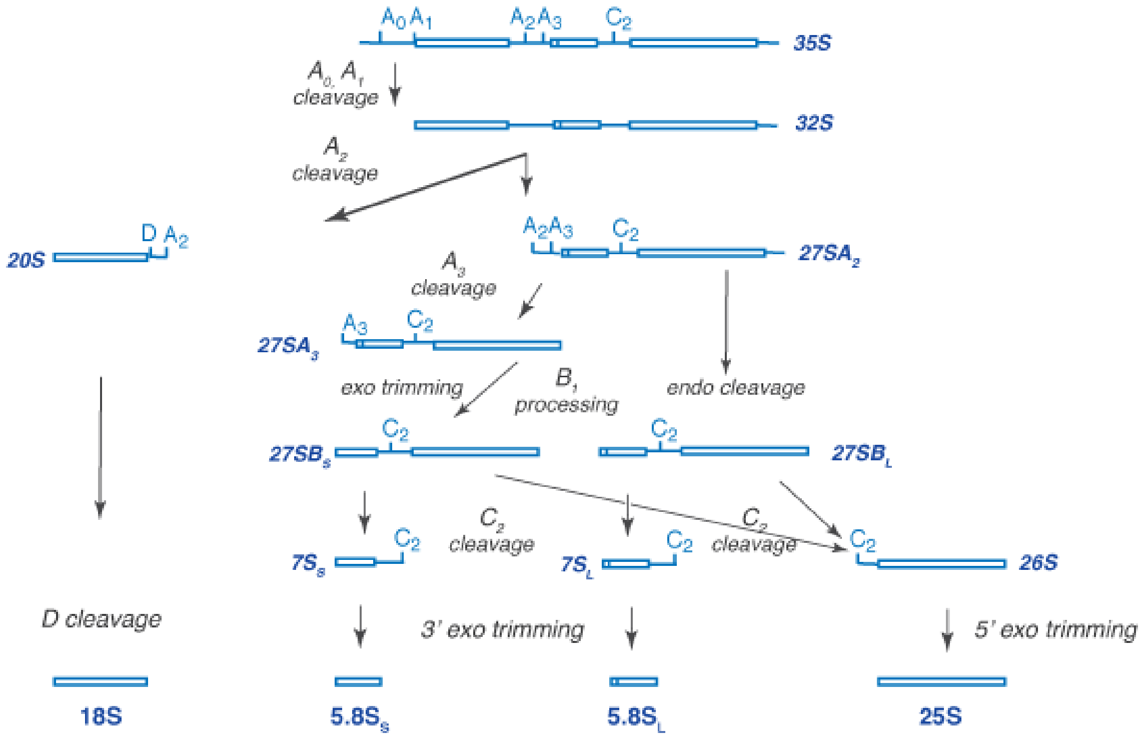

Oeffinger et al.,Supplementary Figure 1.

Figure S1. Diagrammatic Summary of the Pre-rRNA Processing Pathway

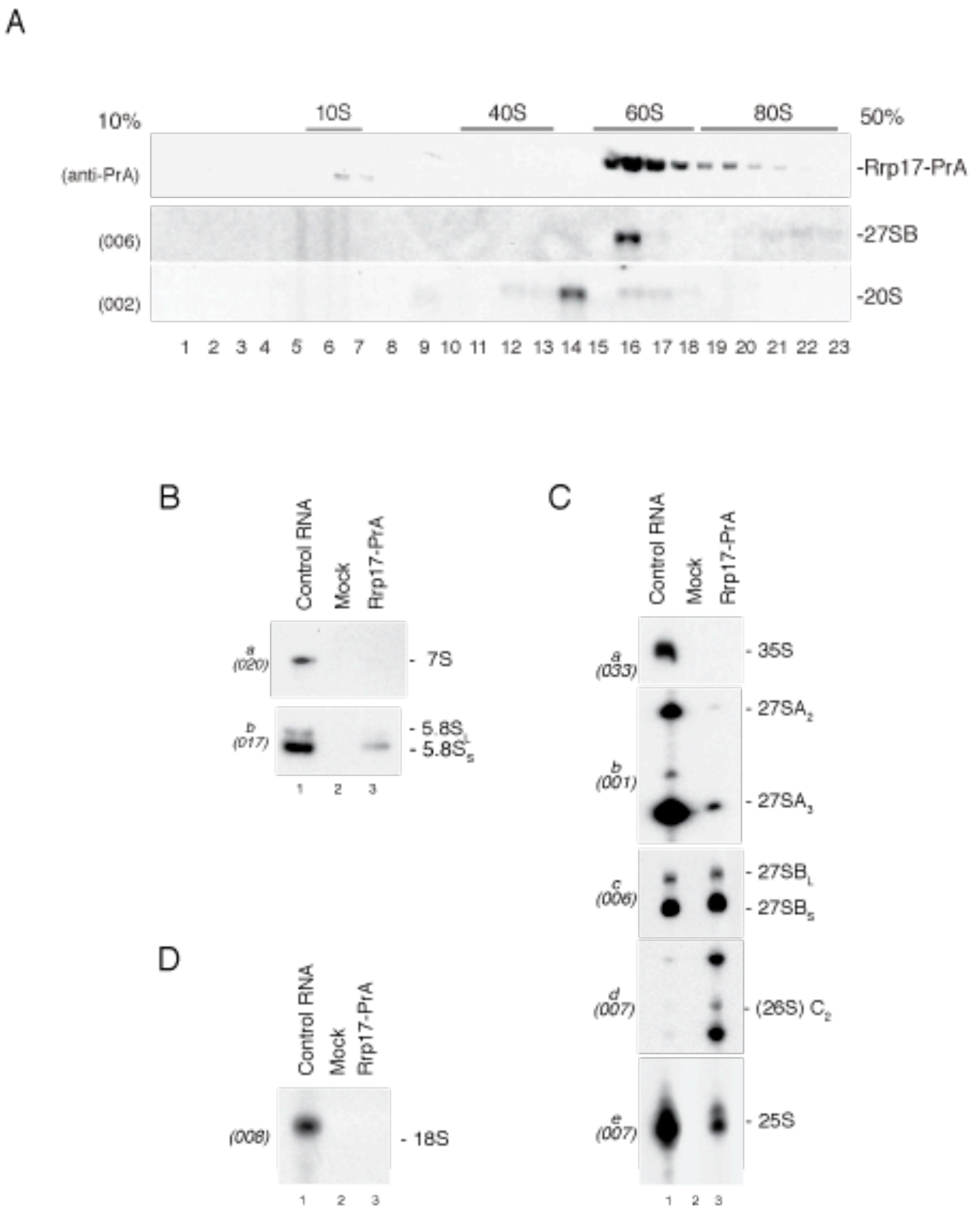

Oeffinger et al.,Supplementary Figure 2.

Figure S2. Rrp17p Is Associated with Pre-60S Ribosomal Subunits

(A) For co-sedimentation analysis lysate from a strain expressing an Rrp17p-PrA fusion was fractionated on a 10-50% sucrose gradient. Fractions were analyzed by Western blotting for the distribution of Rrp17p-PrA and by Northern hybridization for the distribution of the 27SA and 27SB pre-rRNA components of the pre-60S ribosome, and the 20S pre-rRNA component of pre-40S ribosome.

(B-D) RNA co-isolated with Rrp17p-PrA and from a mock-treated, isogenic wild-type control strain was recovered after affinity purification of associated complexes by Proteinase K digest and Phenol/Chloroform extraction. A $1/12^{th}$ of total sample was loaded in each lane. 1µg of total RNA was run for comparison (control RNA). Lane 1: Total RNA control [5µg]. Lane 2: Precipitate from a wild-type control strain. Lane 3: Precipitate from a strain expressing Rrp17p-PrA. (B) Northern hybridization of low molecular weight RNA. (C) Primer extension analyses. (D) Northern hybridization of high molecular weight RNA. Probe names are indicated in parentheses on the left.

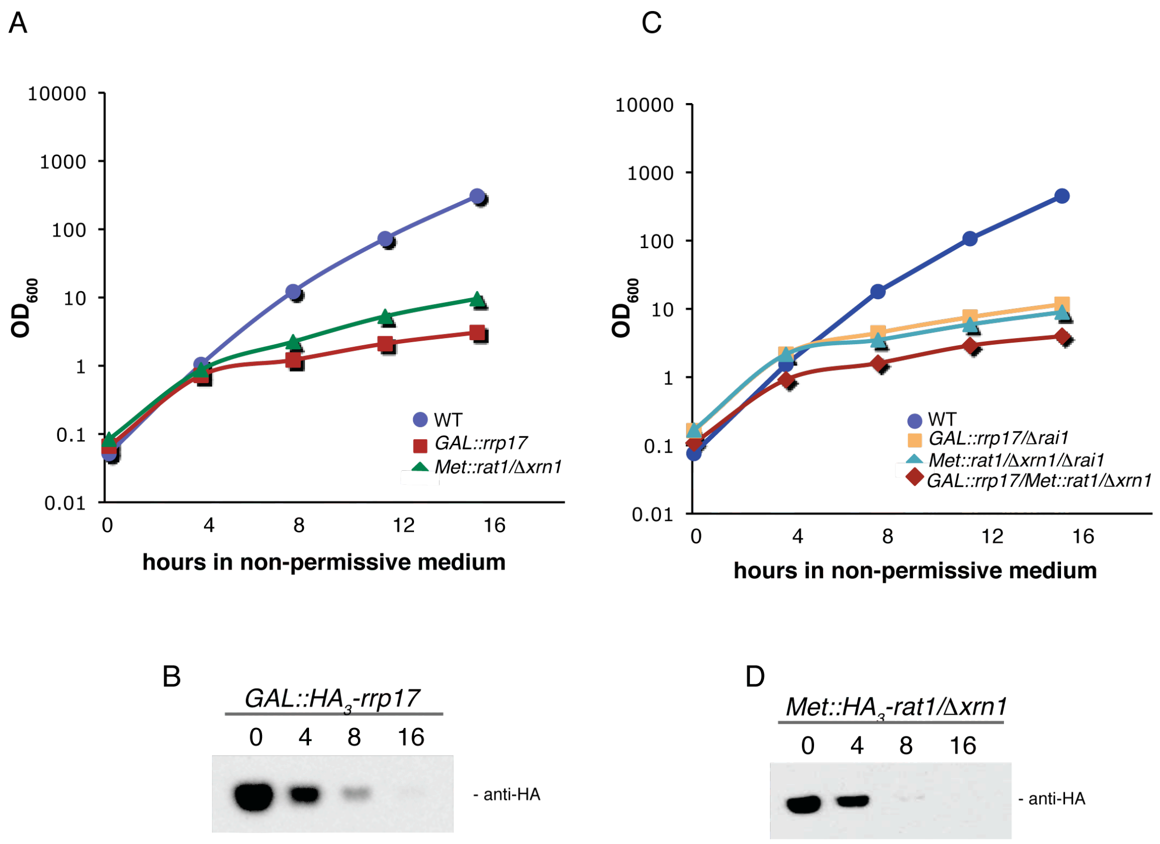

Oeffinger et al., Supplementary Figure 3.

Figure S3. Depletion of Rrp17p and Rat1p

Growth curves of wild-type (λ), *GAL::rrp17* (ν) and *Met::rat1/xrn1Δ* (σ) (A), and wild-type (λ), *GAL::rrp17/rai1Δ* (ν), *Met::rat1/xrn1Δ/rai1Δ* (σ) and *GAL::rrp17/ Met::rat1/xrn1Δ* (υ) (C). Strains were pre-grown in RGS medium or, in the case of *Met::rat1/xrn1Δ*, medium lacking methionine, and transferred to glucose medium with or without 5mM methionine for the times indicated. Strains were maintained in exponential growth by dilution with pre-warmed medium. Cell densities measured by OD_{600} are shown corrected for dilution. Western analyses of the depletion of Rrp17p (B) and Rat1p (D) in *GAL::HA_3-rrp17* and *Met:: HA_3-rat1/xrn1Δ* strains following transfer to glucose- and methionine-containing medium respectively.

A

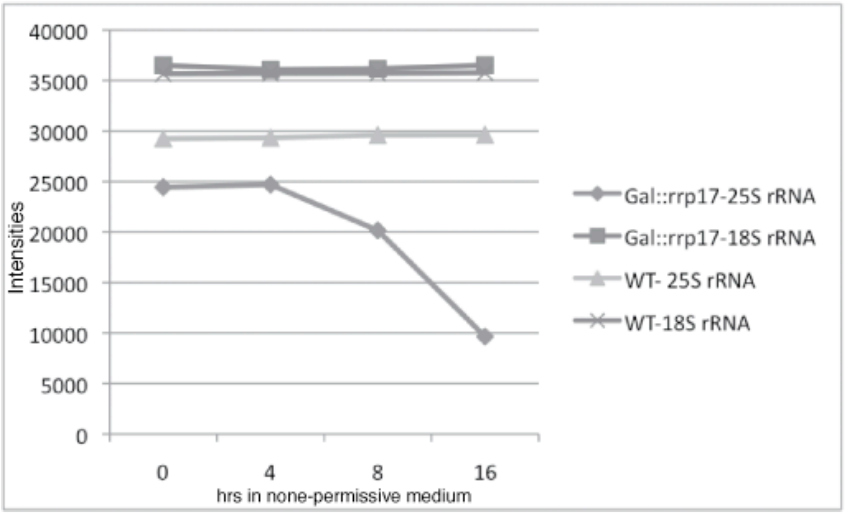

B

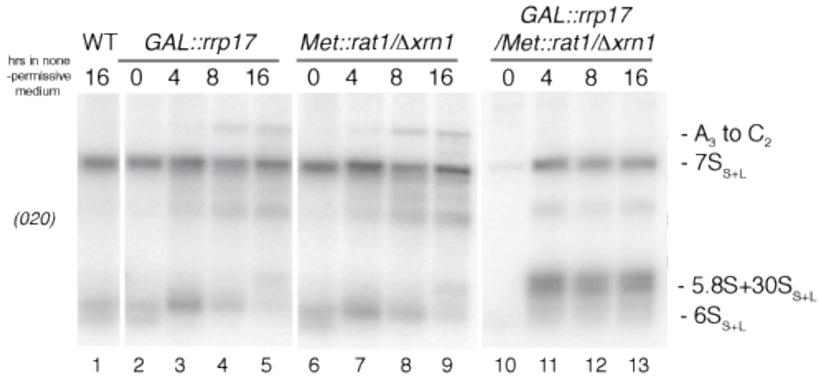

Oeffinger et al., Supplementary Figure 4.

Figure S4. Defects in 5' Exonucleolytic Processing of 5.8S rRNA Effects its 3' End Maturation

(A) Amounts of mature 18S and 25S rRNAs were measured by signal intensities in wild-type and *GAL::rrp17* cells in permissive and non-permissive medium and plotted against each other to determine rRNA processing defects in the absence of Rrp17p.

(B) Northern analysis of high molecular weight RNA. RNA was extracted from wild-type, *GAL::rrp17, Met::rat1/xrn1Δ* and *GAL::rrp17/Met::rat1/xrn1Δ* strains during growth on permissive synthetic complete (SC) or dropout (SD) medium and after transfer to glucose containing medium +/- 5mM methionine for the times indicated.

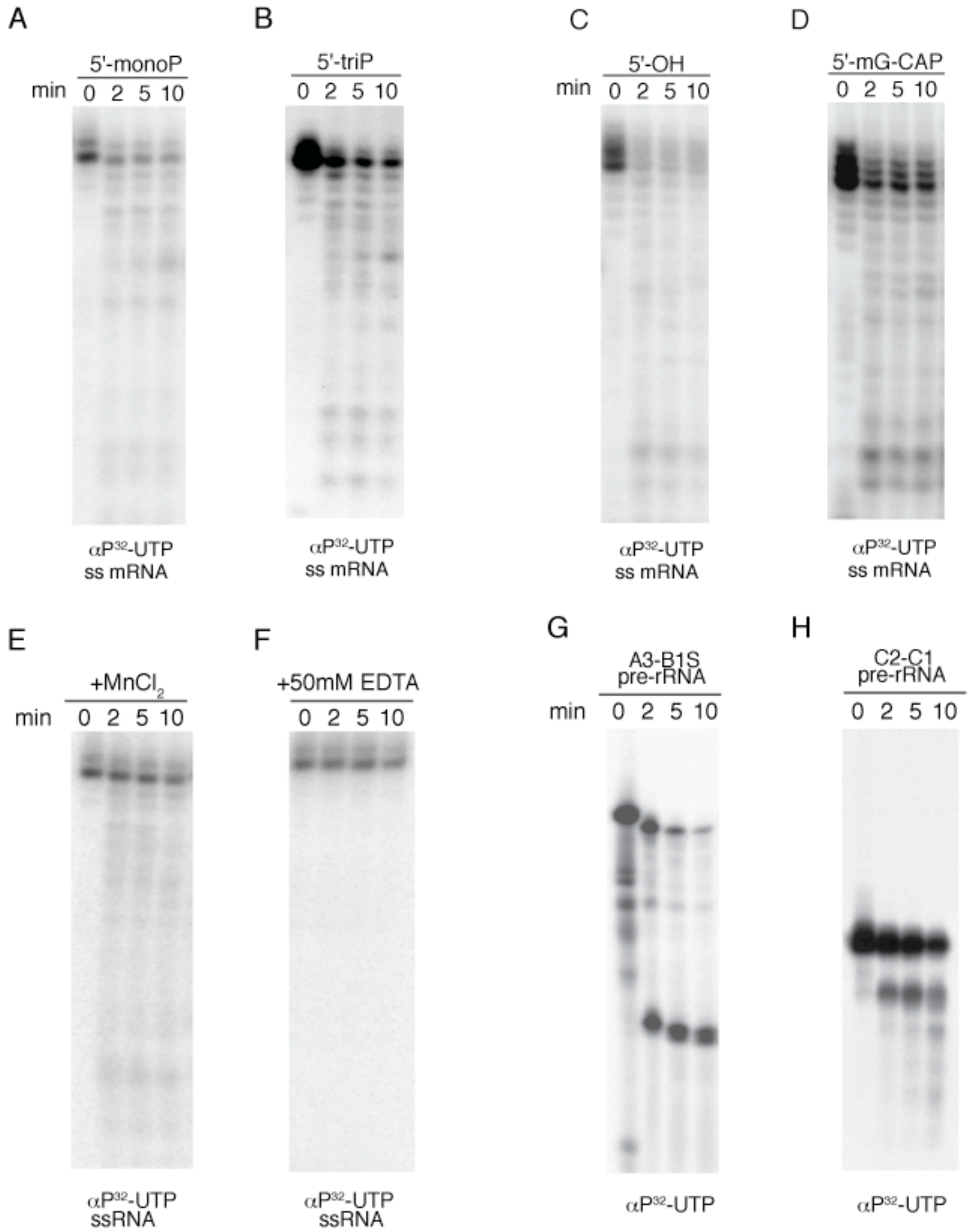

Oeffinger et al., Supplementary Figure 5.

Figure S5. Inhibition of Exonuclease Activity and Degradation of Different rRNA Precursors by Rrp17p

(A-D) A total of 0.25pmol of uniformly labeled (αP^{32}-UTP) RNA was incubated with 50nM His-tagged Rrp17p for the times indicated. Degradation of mRNAs bearing a 5'-monophosphate (5'-monoP) (A) or 5'-triphosphate (5'-triP) (B) group by Rrp17p. Degradation of mRNAs bearing 5'-hydroxyl (5'-OH) group (C) or and 5'-mG cap structure (5'-CAP) (D) by Rrp17p. (E) Degradation of mRNAs by Rrp17p in the presence of (E) $MnCl_2$ and (F) EDTA. RNA was incubated with 50nM His-tagged Rrp17p in the presence of either 1mM $MnCl_2$, replacing $MgCl_2$ in the reaction buffer, or 50mM EDTA for the times indicated. (G, H) Degradation of *in vitro* transcribed pre-rRNAs by Rrp17p. A total of 0.25pmol of uniformly labeled (αP^{32}-UTP), CIP-treated pre-rRNA was incubated with 50nM His-tagged Rrp17p. Nucleic acids were resolved on a 12% acrylamide/urea gels.

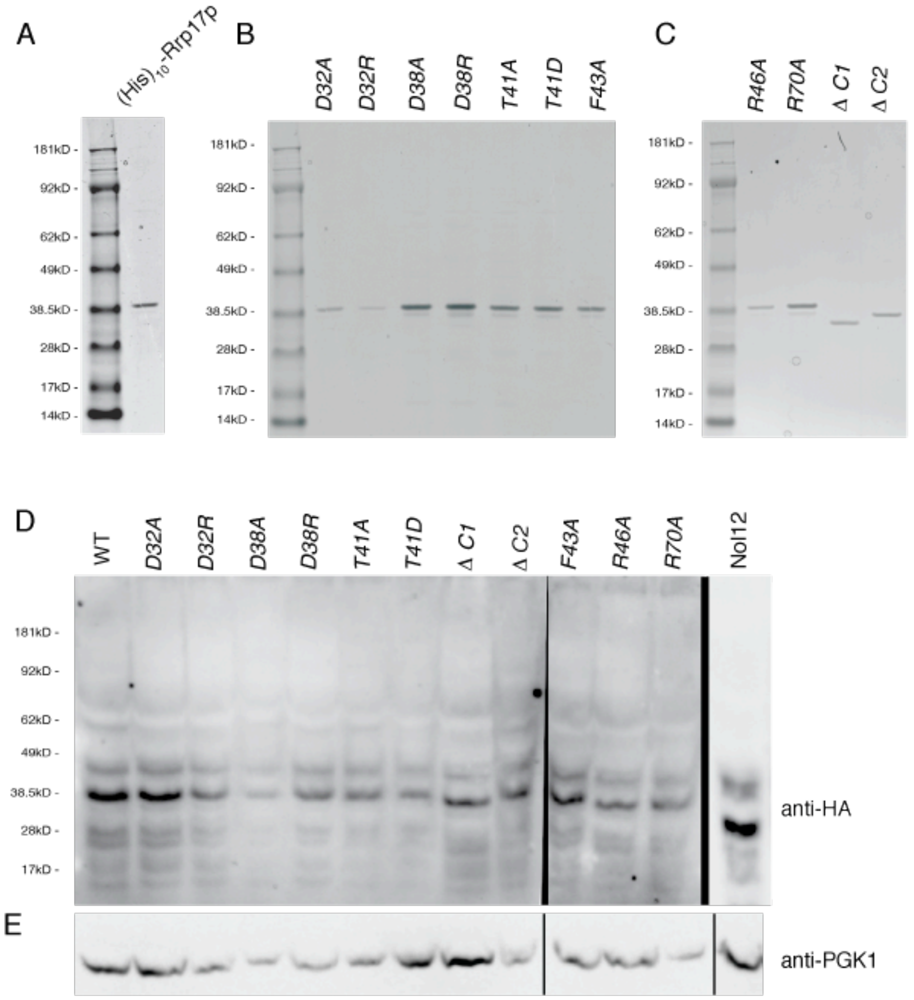

Oeffinger et al., Supplementary Figure 6.

Figure S6. Expression of Recombinant Rrp17p Wild-Type and Mutant Proteins

Rrp17p WT (A) and mutant proteins (B, C) were expressed in *E.coli* as $(His)_{10}$ fusions and eluted from an NTA column using imidazol. (D) Constructs carrying wildtype *RRP17* (pRS414-3xHA-*RRP17*-*TRP*), NOL12 (hRrp17) or different point and truncation mutants were transformed into strains carrying a *RRP17* deletion. Efficient expression of plasmid-expressed wild-type, mutant proteins and the loading control PGK1 (E) were tested by Western Blot analysis using anti-HA and anti-PGK1 antibodies on cell lysates from strains grown at 37°C to mid-log phase.

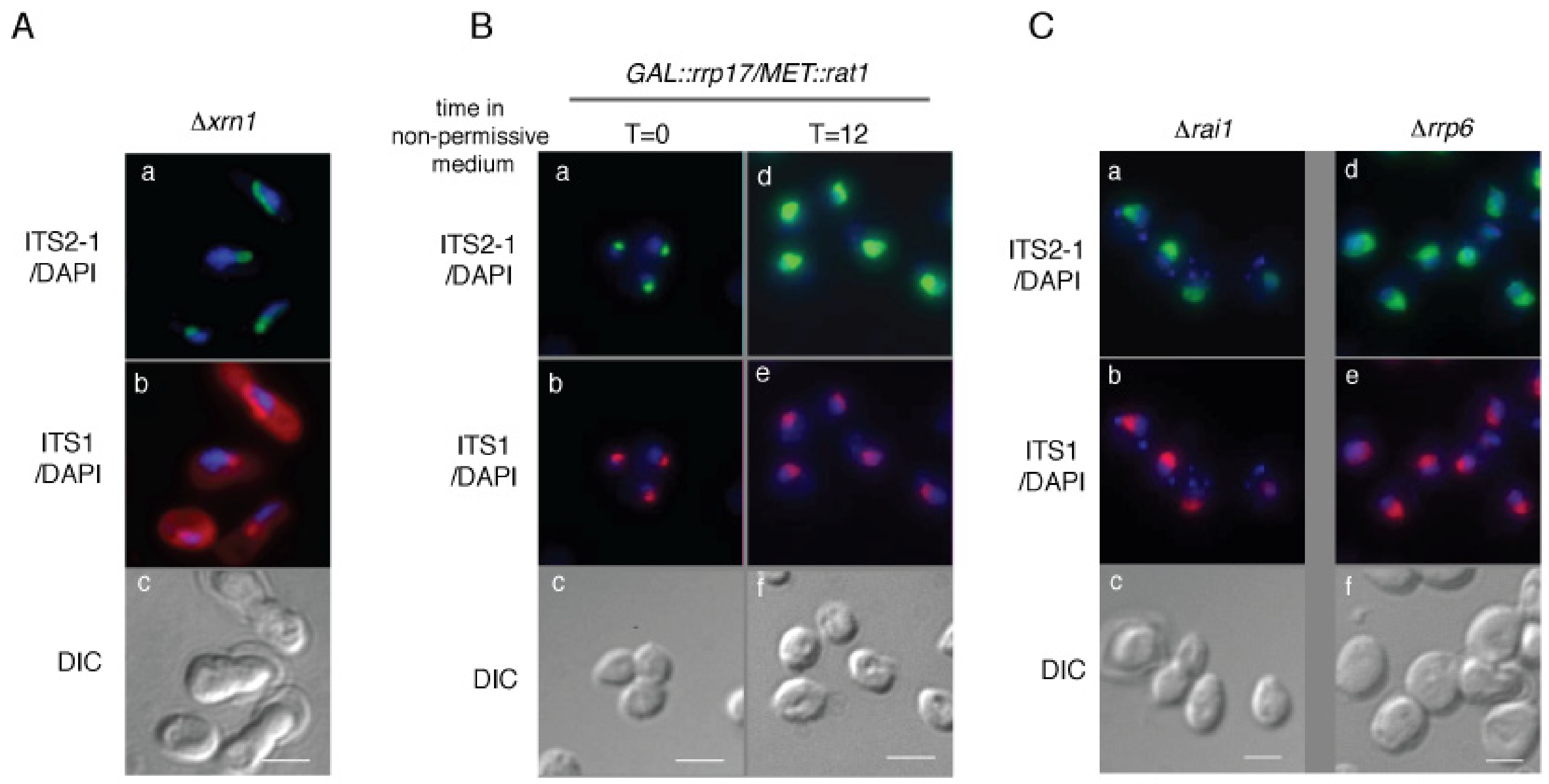

Oeffinger et al.,Supplementary Figure 7.

Figure S7. Export of Pre-60S Subunits Is Delayed by Defects in Late 5’ and 3’ End Processing

Localization of pre-60S (ITS2-1) and pre-40S (ITS1) ribosomal subunits. (A) An *xrn1Δ* strain was grown to mid-log phase. Cells were fixed and mounted with DAPI to stain the nuclei (blue). An increase of 5’ITS1 signal in the cytoplasm was observed in *xrn1Δ* cells, due to the accumulation of excised ITS1 fragments in the absence of Xrn1p. A normal nucleolar distribution of ITS2-1 was observed. (B) *GAL::rrp17/Met::rat1* cells exhibited normal nucleolar distribution of ITS2-1 signal n permissive medium. After 12 hr in non-permissive medium, ITS2-1 was distributed throughout the entire nucleus. In the presence of Xrn1p, no increased ITS1 signal was observed in the cytoplasm, but a normal nucleolar distribution was detected. Bars represent 10μm. (C) Localization of ITS2-1 and ITS1 in *rai1Δ* and *rrp6Δ* cells. The strains were grown to mid-log phase. Cells were fixed and mounted with DAPI to stain the nuclei (blue). In the absence of either Rai1p (left) and Rrp6p (right), cells ITS2-1 was distributed throughout the entire nucleus, while ITS1 exhibited a normal nucleolar distribution. Bars represent 10μm.

Table S1. Yeast Strains Used in this Study

Strain	Genotype	Reference
W303	*Mat a, ade2-1, trp1-1, can1-100, leu2-3, 112, his3-11, 15, ura3Δ, GALpsi+, ssd1-Δ2;*	(Cvrckova and Nasmyth, 1993)
YMO209	*Mat a, ade2-1, trp1-1, can1-100, leu2-3, 112, his3-11, 15, ura3Δ, GALpsi+, ssd1-Δ2,* P_{ZPR1}*::ProteinA::HIS5;*	G. Greco.
YMO197	*Mat a, ade2-1, trp1-1, can1-100, leu2-3, 112, his3-11, 15, ura3Δ, GALpsi+, ssd1-Δ2, RRP17::ProteinA::HIS5;*	This work
YMO223	*Mat a, ade2-1, trp1-1, can1-100, leu2-3, 112, his3-11, 15, ura3Δ, GALpsi+, ssd1-Δ2, GAL::*HA_3*-rrp17-KanMX6*	This work
YAEH110	*Mat a, ade2-1, trp1-1, can1-100, leu2-3, 112, his3-11, 15, ura3Δ, GALpsi+, ssd1-Δ2, Met::rat1-HIS3/xrn1Δ::Nat*	(El Hage et al., 2008)
YMO338	*Mat a, ade2-1, trp1-1, can1-100, leu2-3, 112, his3-11, 15, ura3Δ, GALpsi+, ssd1-Δ2, Met::*HA_3*-rat1-HIS3/xrn1Δ::Nat*	This work
YAEH122	*Mat a, ade2-1, trp1-1, can1-100, leu2-3, 112, his3-11, 15, ura3Δ, GALpsi+, ssd1-Δ2, Met::rat1-HIS3/xrn1Δ::Nat/rai1Δ::KanMX6*	(El Hage et al., 2008)
YAEH120	*Mat a, ade2-1, trp1-1, can1-100, leu2-3, 112, his3-11, 15, ura3Δ, GALpsi+, ssd1-Δ2, rai1Δ::KanMX6*	A. El Hage
YMO339	*Mat a, ade2-1, trp1-1, can1-100, leu2-3, 112, his3-11, 15, ura3Δ, GALpsi+, ssd1-Δ2, Met::rat1-HIS3/xrn1Δ::Nat/GAL::*HA_3*-rrp17-KanMX6*	This work
YMO389	*Mat a, ade2-1, trp1-1, can1-100, leu2-3, 112, his3-11, 15, ura3Δ, GALpsi+, ssd1-Δ2, GAL::*HA_3*-rrp17-KanMX6/rai1Δ::URA3*	This work
YMO404	*Mat α/a, ade2-1, trp1-1, can1-100, leu2-3, 112, his3-11, 15, ura3Δ, GALpsi+, ssd1-Δ2, RRP17/rrp17Δ::KanMX6*	This work
YMO429	*Mat a, ade2-1, trp1-1, can1-100, leu2-3, 112, his3-11, 15, ura3Δ, GALpsi+, ssd1-Δ2, rrp17Δ::KanMX6*, pRRP17-URA3	This work
YMO355	*Mat a, ade2-1, can1-100, leu2-3, 112, his3-11, 15, ura3Δ, RRP17::GFP::HIS5; pNOP1DsRED-LEU2*	This work
YMO356	*Mat a, ade2-1, can1-100, leu2-3, 112, his3-11, 15, ura3Δ, RRP17::GFP::HIS5*	Invitrogen
YMO357	*Mat a, ade2-1, can1-100, leu2-3, 112, his3-11, 15, ura3Δ, NOC1::GFP::HIS5*	Invitrogen
YMO358	*Mat a, ade2-1, can1-100, leu2-3, 112, his3-11, 15, ura3Δ, RRP12::GFP::HIS5*	Invitrogen
YMO359	*Mat a, ade2-1, can1-100, leu2-3, 112, his3-11, 15, ura3Δ, NOC3::GFP::HIS5*	Invitrogen
YMO360	*Mat a, ade2-1, can1-100, leu2-3, 112, his3-11, 15, ura3Δ, DBP5::GFP::HIS5*	Invitrogen

Table S2. Plasmids Used in this Study

Plasmid	Genotype	Reference
pMO105	*pBluescript KS+-AMP*	Stratagene
pMO113	*pRS314-NOP1 DsRED-TRP1*	E. Hurt
pMO213	*pBluescript-rDNA-AMP*	D.Tollervey
pMO401	*pKS132-(HIS)10-RRP17-KAN;*	This work
pMO402	*pKS132-(HIS)10-rrp17 D32A-KAN;*	This work
pMO403	*pKS132-(HIS)10-rrp17 D38A-KAN;*	This work
pMO404	*pKS132-(HIS)10-rrp17 T41A-KAN;*	This work
pMO405	*pKS132-(HIS)10-rrp17 T41D-KAN;*	This work
pMO406	*pKS132-(HIS)10-rrp17 F43A-KAN;*	This work
pMO407	*pKS132-(HIS)10-rrp17 R46A-KAN;*	This work
pMO408	*pKS132-(HIS)10-rrp17 R70A-KAN;*	This work
pMO409	*pKS132-(HIS)10-rrp17 ΔC1-KAN;*	This work
pMO410	*pKS132-(HIS)10-rrp17 ΔC2-KAN;*	This work
pMO411	*pKS132-(HIS)10-rrp17 D32R-KAN;*	This work
pMO412	*pKS132-(HIS)10-rrp17 D38R-KAN;*	This work
pMO414	*pRS414-3xHA-RRP17-TRP1*	This work
pMO415	*pRS414-3xHA-RRP17 D32A-TRP1*	This work
pMO416	*pRS414-3xHA-RRP17 D38A-TRP1*	This work
pMO417	*pRS441-3xHA-RRP17 T41A-TRP1*	This work
pMO418	*pRS414-3xHA-RRP17 T41D-TRP1*	This work
pMO419	*pRS414-3xHA-RRP17 F43A-TRP1*	This work
pMO420	*pRS414-3xHA-RRP17 R46A-TRP1*	This work
pMO421	*pRS441-3xHA-RRP17 R70A-TRP1*	This work
pMO422	*pRS414-3xHA-RRP17 ΔC1-TRP1*	This work
pMO423	*pRS414-3xHA-RRP17 ΔC2-TRP1*	This work
pMO424	*pRS414-3xHA-RRP17 D32R-TRP1*	This work
pMO425	*pRS414-3xHA-RRP17 D38R-TRP1*	This work
pMO426	*pRS416-P_{rrp17} -RRP17-T_{Rrp17}-URA3*	This work
pMO427	*pRS414-3xHA-NOL12(cDNA)-TRP1*	This work
